# P130cas-FAK interaction is essential for YAP-mediated radioresistance of non-small cell lung cancer

**DOI:** 10.1038/s41419-022-05224-7

**Published:** 2022-09-10

**Authors:** Jingduo Li, Xiupeng Zhang, Zaiyu Hou, Siqi Cai, Yingxue Guo, Limei Sun, Ailin Li, Qingchang Li, Enhua Wang, Yuan Miao

**Affiliations:** 1 Department of Pathology, the College of Basic Medical Science and the First Hospital of China Medical University, Shenyang, China; 2 Department of Radiation Oncology, the Shengjing Hospital of China Medical University, Shenyang, China

**Keywords:** Non-small-cell lung cancer, Radiotherapy

## Abstract

Based on the RNA-sequencing data, previous studies revealed that extracellular matrix receptor interaction and focal adhesion signaling pathways were enriched in radioresistant non-small cell lung cancer (NSCLC) cell lines. As the principal members of these signaling pathways, recent studies showed that FAK controlled YAP’s nuclear translocation and activation in response to mechanical activation. However, the underlying mechanisms are largely unknown. This study was designed to determine whether P130cas plays a role in FAK-YAP axis-mediated radioresistance. We found that P130cas promoted proliferation, altered the cell cycle profile, and enhanced tumor growth using cell lines and xenograft mouse models. After treating the cell lines and xenograft models with a single dose of 5 Gy irradiation, we observed that P130cas effectively induced radioresistance in vitro and in vivo. We confirmed that P130cas interacted with and promoted YAP stabilization, thereby facilitating YAP’s activation and nuclear translocation and downregulating the radiosensitivity of NSCLC. Our data also revealed that P130cas and FAK directly interacted with each other and worked together to regulate YAP’s activation and nuclear translocation. Furthermore, the present study identified that P130cas, FAK and YAP formed a triple complex to induce radioresistance. Using P130cas-ΔSH3, FAK- P712/715A mutant, YAP-ΔSH3bm and YAP-ΔWW mutant, our results showed that targeting P130cas-FAK interaction may be a more cost-effective way to overcome the YAP activation mediated radioresistance in NSCLC. Using the data of the public database and our clinical samples, the present study suggested that the expression of P130cas correlated with YAP expression and indicated a poor overall response rate of NSCLC patients who underwent radiation therapy. Overall, our study extends the knowledge of FAK-YAP interaction and provides new insight into understanding the underlying mechanisms to overcome the radioresistance of NSCLC.

## Introduction

Lung cancer continues to be the deadliest human malignancy worldwide with a 5-year overall survival rate of less than 15% [1]. Non-small cell lung cancer (NSCLC) accounts for about 85% of all lung cancer cases [2]. Radiotherapy has been considered a promising and major local treatment strategy for lung cancer. However, local relapse occurs frequently due to radioresistance for most patients [3]. Therefore, there is an urgent need to identify more effective therapeutic targets to enhance lung cancer cells’ radiosensitivity and elucidate the molecular mechanism of radioresistance in lung cancer. The two most recent studies established diverse radioresistant NSCLC cell lines and used RNA-sequencing to study the differentially expressed genes (DEGs) between the radioresistant cells and their parental cells. Functional enrichment analysis of the DEGs based on the Kyoto Encyclopedia of Genes (KEGG) showed that extracellular matrix (ECM)-receptor interaction and focal adhesion signaling pathways were predominantly overexpressed [4, 5]. Cells interact with the surrounding ECM via transmembrane adhesion receptors, which provide a physical link between the ECM and the actin cytoskeleton [6]. Increasing evidence indicates that interactions between the ECM and cancer cells modulate the development of resistance to ionizing radiation [7]. Previous studies revealed that cellular attachment to the ECM induces Yes-associated protein (YAP) nuclear localization and controls transcriptional regulation of cell fates in response to biophysical cues [8]. After translocating into the nucleus, YAP binds with TEAD4 and elevates the transcription of its downstream CTGF and CYR61, thereby accelerating proliferation [9, 10]. Although YAP upregulation and nuclear translocation are frequently observed in clinical samples from cancer patients, genetic alterations of YAP signaling pathway components are rare. The activation of YAP might be a consequence of crosstalk with other signaling pathways [11, 12]. Existing literature suggests that the interaction of ECM proteins and their receptor integrins activates focal adhesion kinase (FAK), leading to cell survival and proliferation [13]. FAK is a key protein within focal adhesions, becoming activated when recruited to focal adhesions after integrin attachment to the ECM [14]. FAK acts as an essential link between a myriad of extracellular signaling inputs and intracellular signaling outputs, with the autophosphorylation of Tyr397 allowing effector proteins to recognize its activated state [15]. A recent study showed that FAK controlled YAP’s nuclear translocation and activation in response to mechanical activation [16]. However, the underlying mechanisms of how the ECM-FAK axis regulates the activation of YAP are largely unknown.

P130cas (also called BCAR1) has been proven to be a component of focal adhesion and is a member of the Cas (Crk-associated substrate) family, which is an adaptor protein that has emerged as a highly connected signaling node [17, 18]. P130cas plays an important role in normal and pathological cell functions. The structure of P130cas is composed of an N-terminal SH3 (Src homology 3) domain, a substrate domain and followed a C-terminal sequence [17, 18]. The interaction between P130cas and FAK at its SH3 domain and binding with Src at its C-terminal binding site is essential for maintaining the integrity and function of focal adhesion [17, 19]. The publishing literature focused on the regulatory functions of P130cas phosphorylation but not P130cas expression. Intriguingly, Tamura et al. demonstrated that overexpression of P130cas increased total tyrosine phosphorylation levels of P130cas without affecting those of FAK [20]. As we know that P130cas is ubiquitously expressed in multiple cancers, and elevated P130cas levels have been associated with poor prognosis in breast cancer, bladder cancer, hepatocellular cancer and lung cancer [21–24]. A previous report has demonstrated that both the phosphorylated FAK and P130cas were elevated after being treated with ionizing irradiation, indicating the implication of FAK and P130cas in regulating radiosensitivity [25]. Although a direct causal link between P130cas expression and radiation therapy resistance has not been reported yet, we hypothesize that P130cas may play a role in FAK-YAP-mediated radioresistance.

This study tested a hypothesis that P130cas may play a role in FAK-YAP axis-mediated radioresistance in NSCLC. For this, we genetically manipulated P130cas expression in lung cancer cells and tested their proliferation and radiosensitivity in vitro and in vivo. We also investigated the interaction between P130cas, FAK and YAP and assessed their functional significance.

## Materials and methods

### Cell lines

Non-small cell lung cancer (NSCLC) cell lines A549, H1299, and H460, and the LLC cell lines were obtained from Shanghai Cell Bank (Shanghai, China). The LK2 cell line was a gift from Dr. Hiroshi Kijima (Department of Pathology and Bioscience, Hirosaki University Graduate School of Medicine, Japan). Cells are stored frozen in individual aliquots. For experiments, all cells were cultured in RPMI 1640 (Invitrogen, Carlsbad, CA, USA) containing 10% fetal calf serum (Invitrogen), 100 IU/mL penicillin (Sigma, St. Louis, MO, USA), and 100 μg/mL streptomycin (Sigma) in sterile culture dishes. For passage, the cells were dissociated by incubation in 0.25% trypsin (Invitrogen); the culture medium was replaced every two or three days; experiments were conducted using cells within ten passages. All cell lines were authenticated by short tandem repeat (STR) DNA profiling.

### Plasmids, shRNA, siRNA and lentivirus

PCDNA3.1-type (WT) YAP-Flag, pCDNA3.1-YAP-WW domain-deletion-mutant (ΔWW)-Flag and pCDNA3.1-YAP SH3bm domain-deletion-mutant (ΔSH3bm)-Flag; pCDNA3.1-Myc-FAK and pCDNA3.1-Myc-FAK P712/715A mutant plasmid were purchased from GenePharma Co., Ltd (Shanghai, China). Short hairpin RNA (shRNA) for YAP and P130cas silencing and respective scrambled control shRNAs were purchased from GeneChem Co., Ltd. (Shanghai, China). FAK-specific and scrambled control siRNAs were purchased from Ribobio (Guangzhou, Guangdong, China). P130cas full-length cDNA plasmid and its ΔSH3 mutant were performed using a lentiviral vector (Ubi-MCS-3FLAG-CBh-gcGFP-IRES-puromycin, GV492, GeneChem). Transfections were done with Lipofectamine 3000 (Thermo Fisher Scientific, Cleveland, OH, USA).

### Western blotting and immunoprecipitation

The assays were performed as previously described [26]. Each experiment was repeated in triplicate. Antibodies against GAPDH were purchased from Sigma and used at 1:5,000 dilutions. Antibodies against P130cas(#13846), p-P130cas (Tyr410, #4011), YAP(#14074 and #12395), p-YAP(#4911), γ-H2AX(#9718), FAK(#71433), p-FAK(Tyr 397,#8556), p-FAK (Tyr925,#3284), Lats1 (#3477), p-Lats1(#8654), cleaved caspase-3(#9664), cleaved PARP(#5625), Lamin B1(#13435), CyclinA2(#67955), CyclinB1(#12231), CyclinD1(#55506), CyclinE1(#20808), Flag (#14793 and #8146) and Myc-tag (#2276 and #2278) were purchased from Cell Signaling Technology (Beverly, MA, USA) and used at 1:1000 dilutions. Antibodies against P130cas (610271) and FAK (610087) (1:1,000) were purchased from BD Transduction Laboratories (Lexington, KY, USA). Antibodies against α-tubulin (1:500) were purchased from Beyotime (Shanghai, China). Verteporfin (12 ng/mL) and PF-562271 (2 μM) were purchased from MedChemExpress (Monmouth Junction, NJ, USA).

### MTT and colony formation assay

The assay was performed as described by Zhang et al. [26]. Each experiment was repeated in triplicate.

### Flow cytometry for cell cycle analysis

Cells (5 × 10^5^) were seeded into 6-cm tissue culture dishes. Twelve hours after seeding, cells were transduced with lentivirus to overexpress P130cas or transfected with P130cas-shRNA, respectively. Forty-eight hours later, cells were harvested, fixed in 1% paraformaldehyde, washed with PBS, and stained with 5 mg/ml propidium iodide (PI) in PBS supplemented with RNase A (Roche, Indianapolis, IN, USA) for 30 min at room temperature. Data were collected using BD systems. A one-parameter histogram was plotted according to nuclear DNA content in each cell as detected by flow cytometry. Cells in each phase of the cell cycle were determined based on DNA ploidy profiles. Each experiment was repeated in triplicate.

### Mouse models

All animal experiments were conducted following the ethical guidelines for treating experimental animals issued at China Medical University and were approved by the Animal Care and Use Committee (KT2020016). Each mouse was randomly assigned to each group and the investigator was blinded to the group allocation. A mouse model of xenografts was established using adult male C57BL/6 mice, which were injected with 2 × 10^5^ LLC cells in the right legs (“primary” or “irradiated” tumor) on day 1. Tumor-bearing mice were sorted into groups according to the mean primary tumor volume before treatment with radiation, which was delivered to primary tumors in three fractions of 8 Gy (8 Gy × 3) on days 10, 11, and 12 after inoculation. Verteporfin was injected intraperitoneally at a dose of 100 mg/kg every two days from day 10 to day 18; vehicle-injected mice were used as control. For locally irradiated primary tumors, mice were placed on a jig, which shielded their entire body except for the leg bearing the targeted tumor, thus allowing a negligible abscopal radiation effect. Tumor size measurement, drug delivery, and sample collection were performed as described in our previous publication [26].

### Linear-quadratic model

Cell survival was quantified using the colony formation assay for dose range from 0 to 8 Gy for the combination of radiation with or without P130cas. Cells were seeded into 6-well plates with cell densities depending on the dose of radiation received; 0–2 Gy: 500 cells, 4 Gy: 1,000 cells, and 8 Gy: 2000 cells. The plating efficiency (PE) was calculated to be the percentage of the cells plated to develop colonies. The value of PE was subsequently used to determine the surviving fraction (SF) for each dose, as the proportion of colonies counted compared with controls. Graphs of radiation dose against surviving fraction were then fitted with the Linear-Quadratic Model (α and β values are the linear and quadratic components of cell killing). Each experiment was repeated in triplicate.

### Immunofluorescence staining

The assay was conducted as described by Zhang et al. [26]. LK2, A549, H1299, H460 and LLC cells were incubated with antibodies against P130cas (1:50), YAP (1:50), and γ-H2AX (1:50). Each experiment was repeated in triplicate.

### Calculation of image colocation coefficient

The fluorescent image is imported into Fiji software and the ROI tool is used to circle the range to be measured to remove the background influence. Then use the co-location threshold of the co-location analysis plug-in built in Fiji software (http://fiji.sc) for co-location analysis. The Rcoloc parameter in the generated result represents the Pearson parameter of the image above the threshold [27].

### Functional enrichment analyses

DAVID (https://david.ncifcrf.gov/summary.jsp), an online tool for gene functional enrichment, was used for gene ontology (GO) analysis (with respect to cellular component, molecular function, and biological process) and the Kyoto Encyclopedia of Genes and Genomes (KEGG) pathway analysis of the 187 DEGs shared between high and low expression of P130cas group. The results were displayed using the ggplot2 R package. *P* < 0.05 was considered statistically significant.

Gene set enrichment analysis (GSEA) was adopted to identify the signaling pathway of elevated P130cas-related gene signature in NSCLC patients. *P* < 0.05 was considered statistically significant. The pathways used for GSEA were obtained from the Molecular Signatures Database (MSigDB) (http://software.broadinstitute.org/gsea/_msigdb).

### RNA extraction and real-time RT-PCR

The protocols are described in a previous publication [26]. Primer sequences are listed in Table 1. Each experiment was repeated in triplicate.

**Table 1:** Primers.

Primer sequences (5′→3′)	
CTGF	5′CAGCATGGACGTTCGTCTG3′ 5′AACCACGGTTTGGTCCTTGG3′
CYR61	5′CTCGCCTTAGTCGTCACCC3′ 5′CGCCGAAGTTGCATTCCAG3′
GAPDH	5′TGTGGGCATCAATGGATTTGG3′ 5′ACACCATGTATTCCGGGTCAAT3′

### Luciferase reporter assay

The assay was performed as previously described [28]. A TEAD reporter plasmid was purchased from Biotime Biotechnology (Shanghai, China), and luciferase activity was measured in cellular extracts using a dual-luciferase reporter gene assay kit (Promega, San Luis Obispo, CA, USA). Each experiment was repeated in triplicate.

### Immunohistochemistry

Immunohistochemistry analysis was conducted as described earlier [26]. Tissue sections were incubated with rabbit monoclonal antibodies against P130cas and YAP (1: 50; Cell Signaling Technology, Danvers, MA, USA). P130cas and YAP staining intensity were scored as 0, 1, 2, and 3, corresponding to no signal or weak, moderate, or high signals, respectively. Two investigators, who were blinded to the clinical data, examined all the of tumor slides. The percentage of stained cells was scored as 1, 1–25%; 2, 26–50%; 3, 51–75%; and 4, 76–100%. The scores of each tumor sample were multiplied to give a final score from 0 to 12; tumors with a score ≥4 were considered as having strong expression, whereas those with a score <4 were considered as having a negative or weak expression of P130cas and YAP.

### Patients and specimens

This study was conducted with the approval of the local institutional review board of China Medical University, and all the patients had signed informed consent. The study was performed in accordance with the Declaration of Helsinki. We also selected 100 (80 male and 20 female) ethnic Han Chinese patients clinically diagnosed as unresectable primary lung carcinoma and pathologically confirmed as NSCLC who underwent radical intensity-modulated radiotherapy (IMRT) at the Radiation Oncology Department of the First Hospital of China Medical University in Northern China between June 2015 and December 2017. Forty-one patients were diagnosed with Squamous cell carcinoma, and 59 were Adenocarcinoma.20 patients were TNM I and II stages, 80 were III and IV stages. All patients received IMRT of radical dose (1.8–2.0 Gy per fraction, totally 56–60 Gy) and repeating CT scan four weeks and 12 weeks after finishing radiotherapy. Target lesions and tumor response were evaluated by Response Evaluation Criteria In Solid Tumors (RECIST) 1.1 [29]. Complete response (CR) and partial response (PR) were regarded as good efficacy, and stable disease (SD) and progressive disease (PD) were defined as poor outcomes in this study. Those (a) with synchronous or metachronous malignant tumors; (b) with distant metastasis found before radiotherapy; (c) who underwent radiotherapy but did not reach radical dose; (d) with incomplete pathological data entries were excluded from this study.

The histological diagnosis and differentiation grade was determined by analyzing hematoxylin/eosin-stained sections according to the WHO classification guidelines (2015) [26].

### Statistical analysis

The data were statistically evaluated using SPSS 23.0 (Chicago, IL, USA). The immunohistochemistry results were analyzed using Spearman’s rank correlation. Differences between groups were compared using Student’s *t* test, and *P* < 0.05 was considered statistically significant.

## Results

### P130cas promotes the proliferation of NSCLC cells in vitro and in vivo

**Fig. 1:**
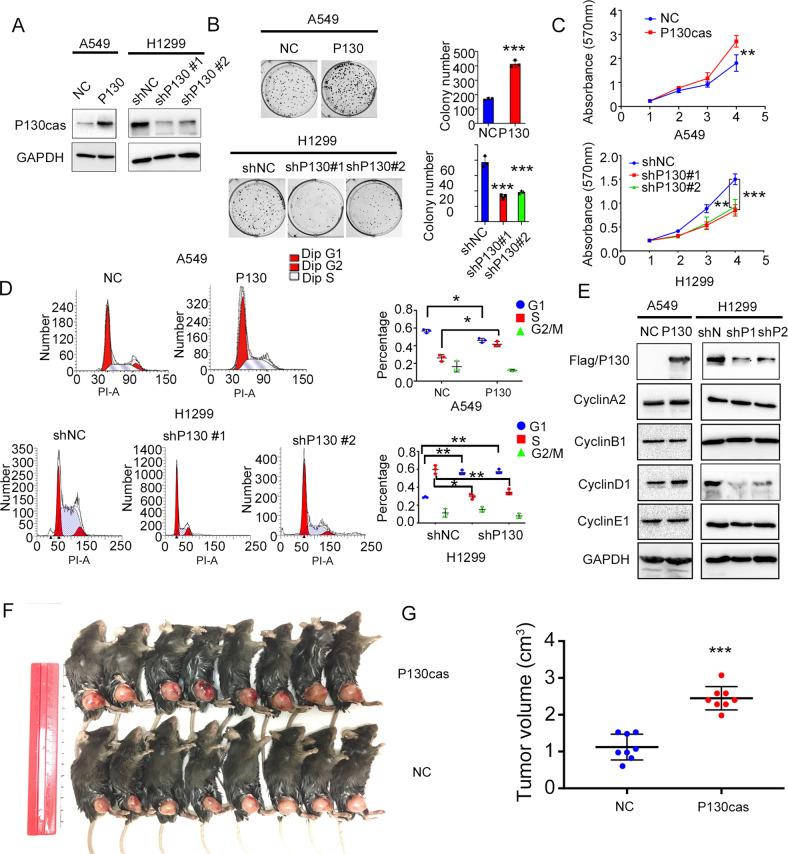
Elevating P130cas enhances the proliferation of NSCLC in vitro and in vivo. **A** Stable up- or down-regulation of P130cas expression by two different shRNAs were confirmed by western blotting in A549 cells and H1299 cells, respectively. Colony formation assay (**B**), MTT assay (**C**), and cell cycle analysis (**D**) using the A549-NC and -P130cas clones, and H1299-NC and –shP130cas#1 and #2 clones. Quantification data are expressed as Mean ± SD of three independent experiments (*t*-test, two-sided, *P < 0.05, **P < 0.01, ***P < 0.001). **E** Western blotting assay detected the expression of cell cycle-related proteins in the A549-NC and -P130cas clones and H1299-NC and –shP130cas #1 and #2 clones (three biological replicates). **F**, **G** Representative examples of explanted tumors in the LLC-P130cas and LLC-NC study group. Quantification data are expressed as Mean ± SD of three independent experiments (*t*-test, two-sided, *P < 0.05, **P < 0.01, ***P < 0.001). For Western blot experiments, the samples derive from the same experiment and the gels/blots were processed in parallel.

We chose A549, H460 and Lewis lung cancer (LLC) cells to establish cell lines stably overexpressing P130cas and H1299 and LK2 cells to establish cell lines with stable P130cas knockdown. The efficiency of lentivirus infection or shRNA transfection was verified by western blotting (Fig. 1A and Supplementary Fig. 1A). As shown in Fig. 1B, C and Supplementary Fig. 1B, C, P130cas overexpression significantly promoted the colony formation abilities and proliferation of A549 and H460 cells compared to controls, whereas P130cas knockdown significantly inhibited the growth of H1299 and LK2 cells. We next assessed their effects on cell cycle progression. Our data revealed that the S phase was visibly prolonged or shortened when P130cas was overexpressed or silenced, respectively (Fig. 1D and Supplementary Fig. 1D). Accordingly, we also evaluated the cell cycle-related proteins expression by western blotting and found that the expression of Cyclin D1, which served in the S phase, was upregulated or downregulated when P130cas overexpression or depletion, respectively (Fig. 1E and Supplementary Fig. 1E). Subsequent xenografts studies also revealed that overexpressing P130cas accelerated LLC cells proliferation in vivo (Fig. 1F, G)

### P130cas induces radioresistance of NSCLC cells in vitro and in vivo

**Fig. 2:**
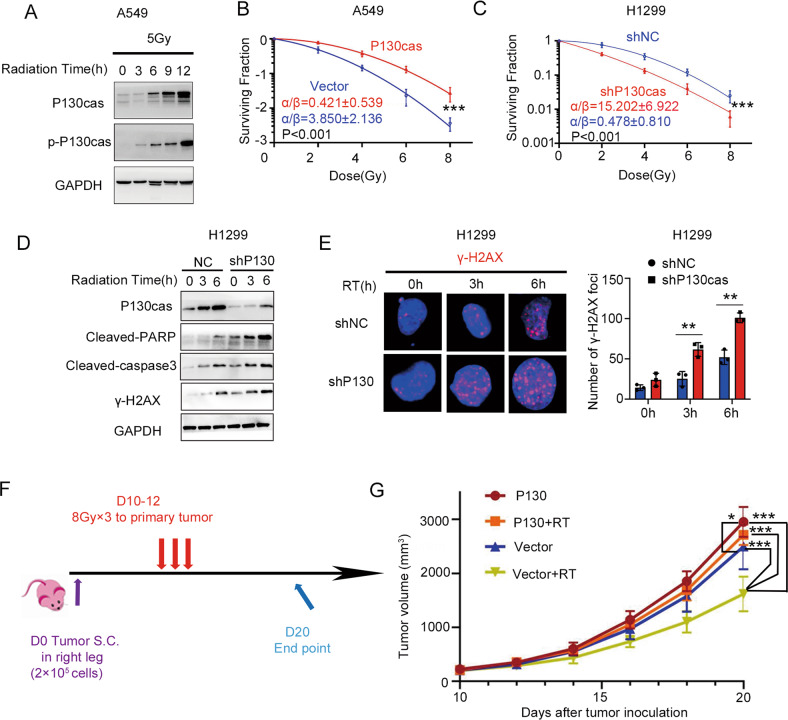
Elevated P130cas induced irradiation-therapy resistance of NSCLC cell lines. **A** P130cas and p-P130cas levels were examined by western blotting at various time points after IR. GAPDH was used as a control. **B**, **C** Clonogenic cell survival curves fitted with the linear-quadratic model for the combination of irradiation with overexpressing or silencing P130cas. Western blotting analysis (**D**) was used to evaluate the expression of cleaved-caspase-3, cleaved PARP and γ-H2AX. Immunofluorescence assay (**E**, scale bar = 10 μm) was used to evaluate the number of γ-H2AX foci at various time points after IR in the H1299-shNC and -shP130cas clones. LLC cells (2 × 10^5^) were subcutaneously injected into mouse legs and irradiated three times on days 10–12 post-injections (*n* = 8); mice were sacrificed on day 20 (**F**). Representative explanted tumor growth curve of mice treated as indicated (**G**, *n* = 8 per group). Each experiment was quantified as Mean ± SD of three independent experiments (*t*-test, two-sided, *P < 0.05, **P < 0.01, ***P < 0.001). For Western blot experiments, the samples derive from the same experiment and the gels/blots were processed in parallel.

Next, we irradiated A549 cells with X-ray (5 Gy) and found that both P130cas and phosphorylated P130cas were continuously upregulated during the post-irradiation period (Fig. 2A). Subsequent analysis of cell survival fractions showed that the P130cas-overexpressing cells were more resistant to radiation than the control group (A549-P130cas *vs* A549-Vector, α/β: 0.421 ± 0.539 *vs* 3.850 ± 2.136; H460-P130cas *vs* H460-Vector, α/β: 0.902 ± 0.819 *vs* 10.613 ± 5.930), whereas the P130cas-depleting cells demonstrated enhanced radiosensitivity (H1299-shP130cas *vs* H1299-shNC, α/β: 15.202 ± 6.922 *vs* 0.478 ± 0.810; LK2-shP130cas *vs* LK2-shNC, α/β: 12.385 ± 1.642 *vs* 3.437 ± 1.227; Fig. 2B, C and Supplementary Fig. 2A, B, Table 2. The western blotting results revealed the upregulation of cleaved PARP and cleaved Caspase-3 after irradiation with a single 5 Gy dose. However, the increase was significantly enhanced by P130cas depletion in H1299 cells and was attenuated by P130cas overexpression in A549 and H460 cells (Fig. 2D and Supplementary Fig. 2C, D). Furthermore, after irradiation, the expression of γ-H2AX (phosphorylation of Ser139 on the H2AX variant), which occurs in response to double-strand DNA breaks and serves as a platform for recruitment of DNA repair mediators, was continuously increased in control H1299, A549 and H460 cells over time. In contrast, the tendency was enhanced or delayed in P130cas-knocking down or -overexpressing cells (Fig. 2D, E and Supplementary Fig. 2C–F). The levels of γ-H2AX in irradiated LLC cells were also decreased by P130cas overexpression (Supplementary Fig. 2G, H). Similar results were obtained in vivo that the xenografts derived from cancer cells overexpressing P130cas showed increased resistance to X-ray irradiation compared with the control group (Fig. 2F, G).

**Table 2:** Parameter of radiobiology fitted linear quadratic model.

	α	95% CI(α)	β	95% CI(β)	α/β
A549 NC	0.234	0.108 to 0.353	0.062	0.025 to 0.114	3.853
A549 P130cas	0.023	−0.036 to 0.080	0.054	0.039 to 0.0712	0.421
H1299 shNC	0.027	−0.065 to 0.115	0.057	0.034 to 0.085	0.478
H1299 shP130	0.395	0.326 to 0.458	0.026	0.007 to 0.050	15.2
H460 NC	0.315	0.218 to 0.403	0.030	0.005 to 0.064	10.61
H460 P130cas	0.042	−0.032 to 0.112	0.047	0.029 to 0.068	0.902
LK2 shNC	0.119	0.070 to 0.168	0.035	0.023 to 0.048	3.437
LK2 shP130	0.378	0.354 to 0.397	0.030	0.023 to 0.038	12.38

### P130cas interacts with and promotes stabilization of YAP, thus inducing radioresistance

**Fig. 3:**
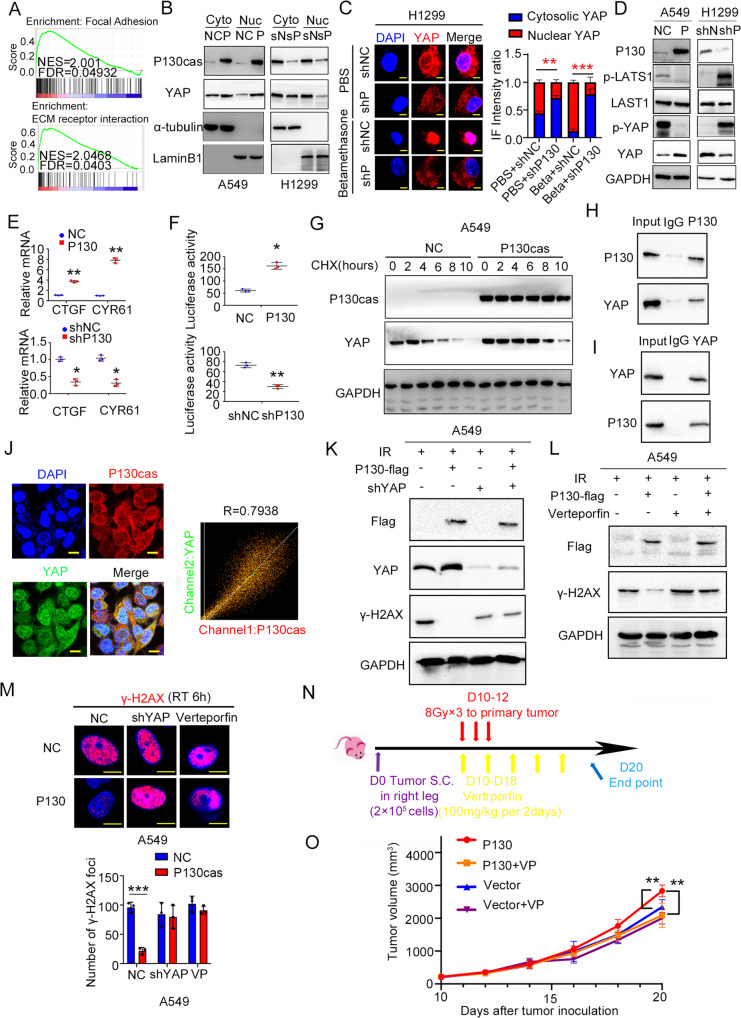
P130cas interacts with YAP and promotes its stabilization, thus inducing radioresistance. **A** GSEA analysis was performed to explore the signaling pathway positively correlated with high P130cas expression in NSCLC. **B** Cytosolic and nuclear proteins were extracted respectively to check YAP expression in the A549-NC and -P130cas clones, and H1299-NC and –shP130cas clones, α-tubulin or LaminB1 were used as the cytosolic or nuclear protein internal control, respectively. In the H1299-NC and –shP130cas clones with or without betamethasone, immunofluorescence staining was used to evaluate the subcellular localization of YAP (**C**, scale bar = 10 μm), western blot assay was used to detect the phosphorylation of LATS1 and YAP (**D**), qPCR assay was used to investigate the alteration of the target genes of YAP (**E**), luciferase reporter assay was used to identify binding activity between YAP and TEAD4 promoter (**F**). After being treated with CHX at indicated time point, the expression of YAP was evaluated by western blotting in the A549-NC and -P130cas clones (**G**). The co-IP assay was used to confirm the interaction between P130cas and YAP (**H**, **I**). Immunofluorescence assay revealed the co-localization of endogenous P130cas and YAP in A549 cells, colocation coefficient was quantified by Fiji software (**J**, scale bar = 10 μm). In the A549-NC and -P130cas clones irradiated by 5 Gy X-ray, after incorporation with YAP-siRNA or Verteporfin, western blotting (**K**, **L**) and immunofluorescence (**M**) were used to observe the expression of YAP and γ-H2AX, as well as the number of γ-H2AX foci (scale bar = 10 μm). Mice were subjected to radiation (8 Gy) on days 10, 11, to 12 after tumor cell inoculation, and verteporfin (100 mg/kg) was injected intraperitoneally every two days from day 10 to day 18 (*n* = 8). Mice were euthanatized on day 20 and analyzed for tumor size (**N**). Representative explanted tumor growth curve of mice treated as indicated (**O**, *n* = 8 per group). Each experiment was quantified as average ± SD of three independent experiments (*t*-test, two-sided, *P < 0.05, **P < 0.01, ***P < 0.001). For Western blot experiments, the samples derive from the same experiment and the gels/blots were processed in parallel.

To provide a deeper understanding of the biological functions of the DEGs between high and low P130cas expressing clinical tissue samples, we performed KEGG and GO pathway enrichment analyses and Gene Set Enrichment Analysis (GSEA). The pathway enrichment analysis results revealed that Focal adhesion (FAs) and ECM receptor interaction processes were the main deregulated pathways between the high and low P130cas expressing group (Fig. 3A and Supplementary Fig. 3A, B). P130cas was a critical factor in modulating FAs by interacting with FAK. It has been demonstrated that YAP, which played a pivotal role in ECM remodeling, was an essential inducer during radioresistance [30–34]. We, therefore, wanted to investigate whether P130cas modulated proliferation and radioresistance by affecting YAP. Using nucleoplasmic separation and immunofluorescence assays, we found that P130cas overexpression significantly promoted the nuclear translocation of YAP, whereas P130cas knockdown significantly inhibited this process (Fig. 3B and Supplementary Fig. 4A). As described by a previous study [35], we chose betamethasone as an acute stimulus to promote YAP nuclear translocation, the immunofluorescent results indicated that P130cas depletion effectively attenuated the nuclear YAP induced by betamethasone treatment (Fig. 3C and Supplementary Fig. 4B). Since the Hippo signaling pathway regulates YAP nuclear transport through its phosphorylation at Ser127 residue [36], using the data from the GEPIA database, we thus evaluated associations between the expression of P130cas and the expression of putative members of the Hippo signaling pathway. The results revealed that P130cas expression was significantly correlated with most members of the Hippo signaling pathway (Supplementary Fig. 4C).

Further western blotting results indicated that the phosphorylated Lats1 and YAP levels were decreased in P130cas-overexpressing A549 and H460 cells and increased in P130cas-depleted H1299 and LK2 cells (Fig. 3D and Supplementary Fig. 4D). Consistent with these observations, the mRNA levels of downstream target genes of YAP (*CTGF* and *CYR61*) and the activity of the TEAD4 luciferase reporter were upregulated or downregulated after P130cas overexpression or knockdown, respectively (Fig. 3E, F and Supplementary Fig. 4E, F). We used cycloheximide (CHX) to block the de novo protein synthesis and investigated the impact of P130cas overexpression on the stability of YAP. We found that the degradation of YAP was inhibited in P130cas-overexpressing A549 and H460 cells and was upregulated in P130cas-depleted H1299 cells (Fig. 3G and Supplementary Fig. 4G). As is known to us all, P130cas is an adaptor protein. Therefore, we may wonder whether it may stabilize YAP by interacting with each other. The results of co-immunoprecipitation (co-IP) and immunofluorescence assays showed that P130cas directly interacted with YAP and co-localized with YAP in the cytoplasm which was quantified by Fiji software (*R* = 0.7938, Fig. 3H–J).

Furthermore, in the cells with stable P130cas overexpression, the upregulation of YAP was inhibited by transfection with YAP-specific shRNA or treatment with Verteporfin (an inhibitor that specifically blocks YAP to activate its downstream target TEAD4). After being treated with a single dose of irradiation (5 Gy), we found that YAP inhibition rescued the repression of γ-H2AX, in both the expression level of γ-H2AX and the number of γ-H2AX foci, caused by P130cas overexpression (Fig. 3K–M and Supplementary Fig. 4H–J). In addition, using mice xenograft models, we confirmed that the incorporation of Verteporfin effectively reversed the increasing growth of xenografts arising from P130cas overexpression (Fig. 3N, O).

### The P130cas-FAK axis regulates YAP activation and contributes to the radioresistance of NSCLC

**Fig. 4:**
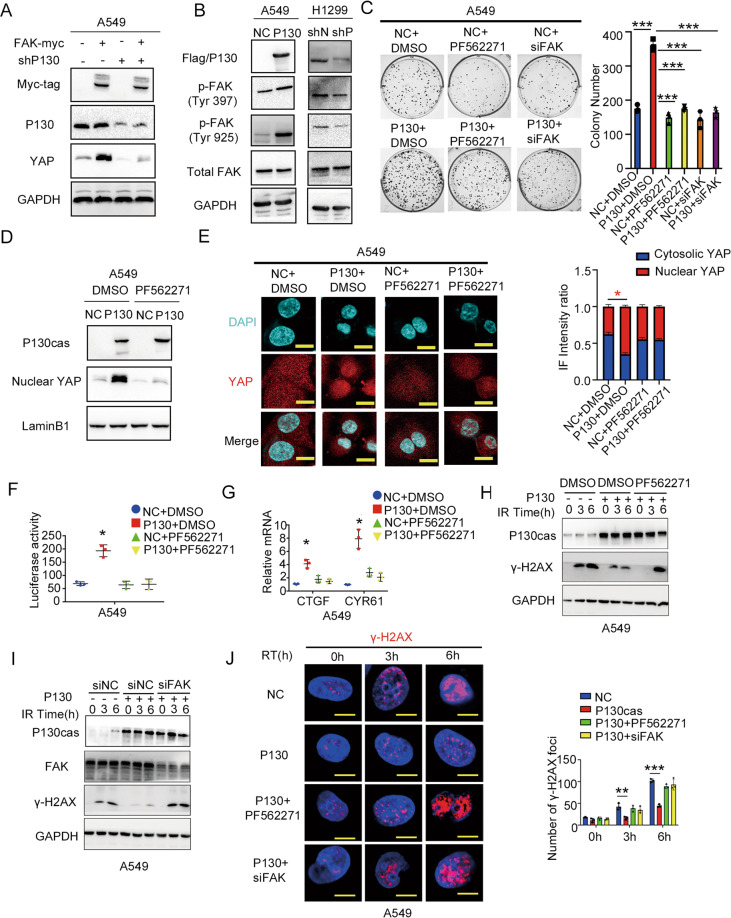
P130cas stabilizes YAP by activating FAK signaling. **A** Immunoblotting of Myc-tag, P130cas (P130), YAP and GAPDH in H1299 cells transfected with FAK-myc alone or in combination with P130cas shRNA. **B** Immunoblotting of Flag/P130cas (P130), FAK-Tyr397, FAK-Tyr925, total FAK and GAPDH in A549 cells overexpressing P130cas or in H1299 cells transfected with P130cas shRNA. **C** Representative images and data quantification of colony formation by A549 cells overexpressing P130cas alone versus in combination with PF562271 or FAK siRNA. **P < 0.01. In A549 cells overexpressing P130cas alone or in combination with PF562271, after nucleoplasmic separation, using immunoblotting to evaluate P130cas, nuclear YAP and LaminB1 (**D**), using immunofluorescence to evaluate YAP subcellular distribution (**E**), using luciferase reporter assay (**F**) and qPCR assay (**G**) to detect the downstream gene activity of YAP (*t* test. *P < 0.05). In A549 cells overexpressing P130cas alone or in combination with PF562271/FAK-siRNA, immunoblotting of P130cas, γ-H2AX and GAPDH at the indicated time points after 5 Gy ionizing radiation (**H**, **I**), representative immunofluorescence images of the number of γ-H2AX foci at the indicated time points after 5 Gy ionizing radiation (**J**, scale bar = 10 μm). Each experiment was quantified as Mean ± SD of three independent experiments (*t*-test, two-sided, *P < 0.05, **P < 0.01, ***P < 0.001). For Western blot experiments, the samples derive from the same experiment and the gels/blots were processed in parallel.

Since P130cas is the putative downstream molecule of FAK and FAK has been proven to promote YAP activation, we next investigated whether FAK activated YAP through regulating P130cas [37]. We found that overexpression of FAK promoted the nuclear expression of YAP, while co-transfection of P130cas shRNA effectively attenuated the increasing level of nuclear YAP arising from FAK overexpression in H1299 cells and LK2 cells (Fig. 4A and Supplementary Fig. 5A). In addition, both overexpressing P130cas and transfecting P130cas shRNA prominently impacted the phosphorylation of FAK in Tyr 397 and Tyr925 (Fig. 4B and Supplementary Fig. 5B). The colony formation assay showed that the increased proliferation of A549 and H460 cells induced by P130cas overexpression was suppressed by inhibiting FAK signaling with PF-562271 or FAK siRNA (Fig. 4C and Supplementary Fig. 5C). Incorporation of PF-562271 or FAK siRNA attenuated nuclear accumulation and activation of YAP arising from P130cas overexpression (Fig. 4D–G and Supplementary Fig. 5D–G). Blocking FAK also decreased the phosphorylation of both Lats1 and YAP (Supplementary Fig. 5H). Correspondingly, inhibition of FAK with PF-562271 or FAK siRNA rescued the repression of γ-H2AX expression evoked by P130cas overexpression after 5 Gy irradiation (Fig. 4H–J and Supplementary Fig. 5I–K).

### P130cas, FAK and YAP formed a triple complex to induce radioresistance

**Fig. 5:**
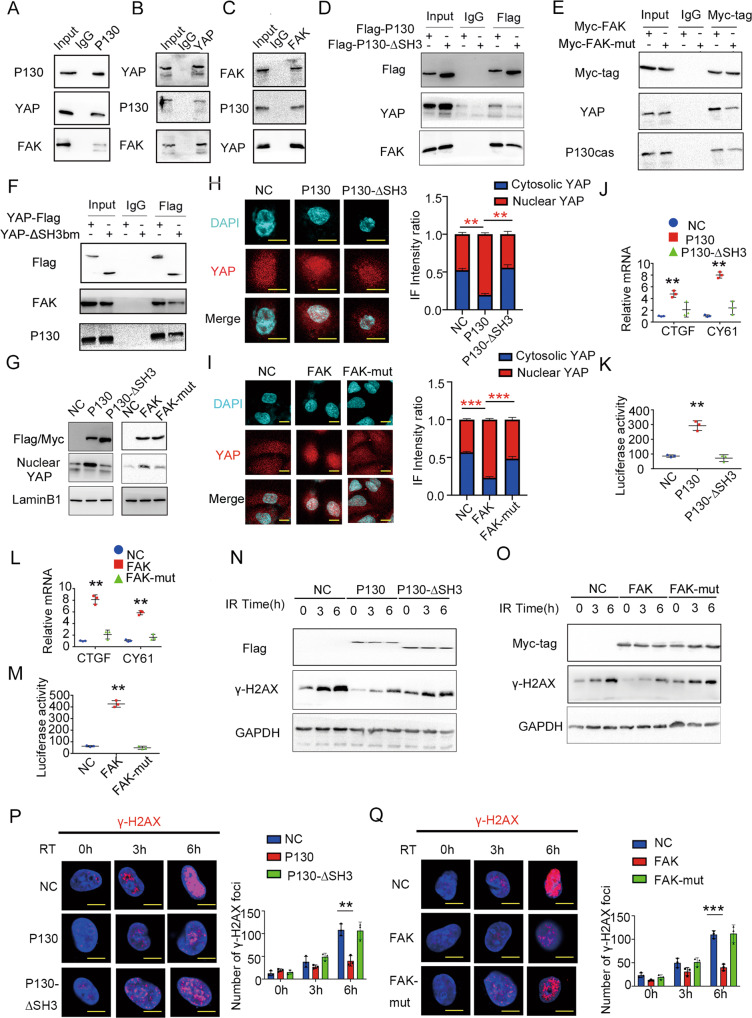
Interaction with P130cas is pivotal in forming the triple complex with FAK and YAP. **A** P130cas was immunoprecipitated from A549 cells and immunoblotted with indicated antibodies. **B** YAP was immunoprecipitated from A549 cells and immunoblotted with indicated antibodies. **C** FAK was immunoprecipitated from A549 cells and immunoblotted with indicated antibodies. **D** P130cas was immunoprecipitated by Flag antibody from A549 cells infected with Flag-P130cas lentivirus vector or Flag-P130-ΔSH3 lentivirus vector and immunoblotted with indicated antibodies. **E** FAK was immunoprecipitated by Myc antibody from A549 cells transfected with Myc-FAK or Myc-FAK-mut (712/715A) and immunoblotted with indicated antibodies. **F** YAP was immunoprecipitated by Flag antibody from A549 cells transfected with Flag-YAP or Flag-YAP-ΔSH3bm and immunoblotted with indicated antibodies. In A549 cells overexpressing Flag-P130cas/Flag-P130-ΔSH3 or overexpressing Myc-FAK/Myc-FAK-mut (712/715A), after nucleoplasmic separation, using immunoblotting to evaluate P130cas/FAK, nuclear YAP and LaminB1 (**G**), using immunofluorescence (**H**, **I**) to identify the subcellular distribution of YAP, using qPCR assay (**J**, **L**) and luciferase reporter assay (**K**, **M**) to detect the downstream gene activity of YAP (*t* test. *P < 0.05). In A549 cells overexpressing Flag-P130cas/Flag-P130-ΔSH3 or overexpressing Myc-FAK/Myc-FAK-mut (712/715A), using immunoblotting to evaluateγ-H2AX and GAPDH at the indicated time points after 5 Gy ionizing radiation (**N**, **O**), representative immunofluorescence images of the number of γ-H2AX foci at the indicated time points after 5 Gy ionizing radiation (**P**, **Q**, scale bar = 10 μm). Each experiment was quantified as Mean ± SD of three independent experiments (*t*-test, two-sided, **P < 0.01, ***P < 0.001). For Western blot experiments, the samples derive from the same experiment and the gels/blots were processed in parallel.

Previous studies demonstrate that P130cas directly binds with the proline-rich region of FAK through its SH3 domain [17, 19, 38]. YAP also has a proline-rich region that is predicted to bind with signaling molecules containing SH3 domains and a WW domain that is predicted to bind with the proline-rich region [39]. To clarify the relationship among P130cas, FAK and YAP, the interaction between P130cas, FAK and YAP was evaluated with co-IP. Our results confirmed that the endogenous P130cas, FAK, and YAP formed a triple complex (Fig. 5A–C). Subsequently, we constructed truncated mutants for P130cas (SH3 domain deletion, P130cas-ΔSH3) and YAP (deletion of SH3 binding motif, YAP-ΔSH3bm; WW domain deletion, YAP-ΔWW), as well as a P712/715A mutant for FAK (FAK-Mut). The results of co-IP suggested that P130cas-ΔSH3 mutant, FAK-Mut mutant and YAP-ΔSH3bm effectively attenuated the formation of P130cas-FAK-YAP complex than the corresponding full-length plasmids, whereas YAP-ΔWW mutant did not show visible impaction on the formation of the triple complex (Fig. 5D, F, Supplementary Fig. 6A). Compared with the cells overexpressing full-length P130cas or FAK, the nuclear accumulation of YAP, expression of *CTGF* and *CYR61*, and the activity of the TEAD4 luciferase reporter were decreased in cells transduced with the P130-ΔSH3 mutant or transfected with FAK P712/715A mutant, respectively (Fig. 5G–M). Accordingly, after a single dose of irradiation (5 Gy), the attenuation of γ-H2AX arising from full-length P130cas and full-length FAK overexpression was also abolished by P130-ΔSH3 mutant lentivirus delivering or FAK-Mut mutant transfection, respectively (Fig. 5N–Q).

### P130cas expression significantly correlated with YAP expression in human lung cancer tissues and radiation therapy output

**Fig. 6:**
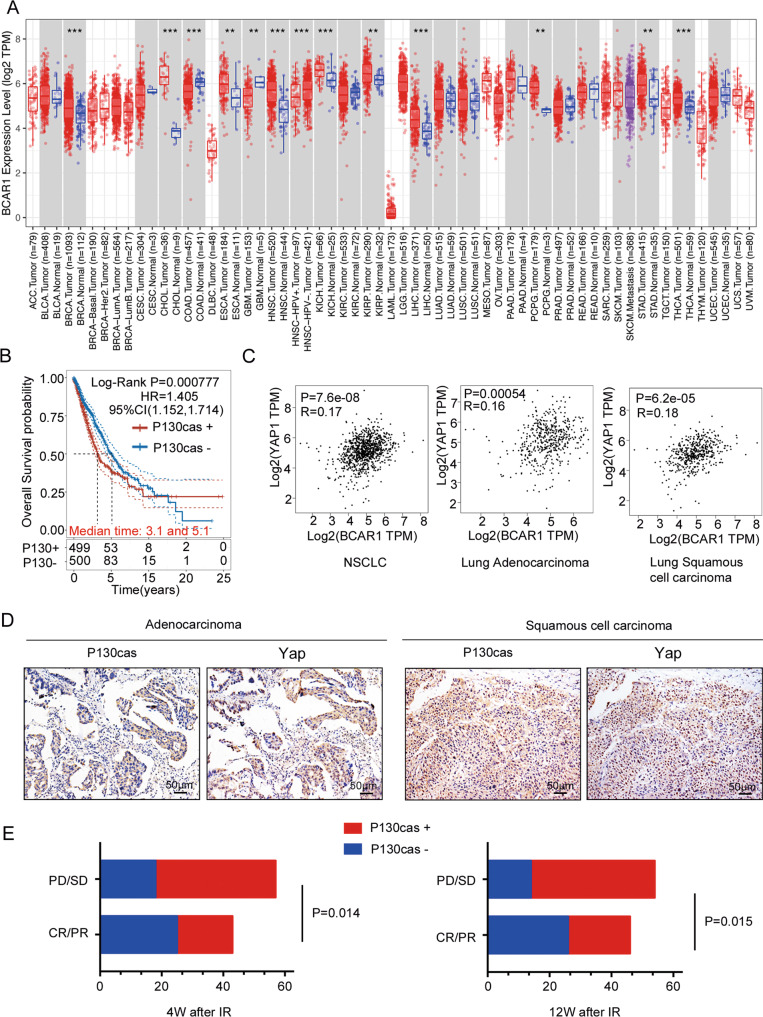
P130cas expression correlated with YAP as well as worse radio-therapy responses in human NSCLC samples. **A** Pan-cancer expression analysis illustrated the universal expression of P130cas in multiple malignant tumor types. **B** Kaplan–Meier analysis using the data from TCGA database showed a correlation with P130cas expression and NSCLC patient survival time. **C** GEPIA database analysis showed the correlation of mRNA levels between P130cas and YAP in NSCLC. **D** Representative images of immunohistochemistry staining of P130cas and YAP in the same NSCLC patients, scale bar: 50 μm. **E** Quantification data using Spearman’s rank correlation demonstrated a correlation between the expression of P130cas and different radiotherapy responses (PR/CR or PD/SD) after 4weeks (left panel) or 12 weeks (right panel). Quantification data are expressed as Mean ± SD of three independent experiments (*t* test. *P < 0.05, **P < 0.01, ***P < 0.001).

Based on the data of mRNA expression profiles from The Cancer Genome Atlas (TCGA) and GTEx, we investigated the expression of P130cas in pan-cancer and normal lung tissue. Although not significant, P130cas showed elevated expression levels in lung adenocarcinoma (LUAD) samples and lung squamous cell carcinoma (LUSC) samples than in non-cancerous lung tissue samples (Fig. 6A). Clinically, high P130cas expression in NSCLC is associated with poor prognosis in TCGA datasets (Fig. 6B). Using the gene expression profiling interactive analysis (GEPIA), we found that the expression of P130cas significantly correlated with the expression of YAP (Fig. 6B). Analysis of our NSCLC samples by immunohistochemistry confirmed that P130cas expression was significantly associated with nuclear YAP levels (*P* < 0.001, *r* = 0.39, Fig. 6D and Table 3). To assess the association of the P130cas expression with response to radiation therapy, we further evaluated the relationship between P130cas expression and treatment response of radiation therapy in the same cohort. We found that P130cas expression significantly correlated with poor overall response rate (ORR). Four weeks after irradiation, in the patients with positive P130cas expression, the ORR was 31.6%, whereas, in the patient with negative P130cas expression, the ORR was 58.1% (*P* = 0.014). Twelve weeks after irradiation, in the patients with positive P130cas expression, the ORR was 35.1%, whereas, in the patient with negative P130cas expression, the ORR was 60.5% (*P* = 0.015, Fig. 6E and Table 4).

**Table 3:** Correlation of P130cas with the expression of YAP in 100 NSCLC specimens.

		P130cas		*r*	*P*
		Negative	Positive		
YAP	Negative	32	20	0.39	<0.001
	Positive	11	37

**Table 4:** Correlation of the expression of P130cas with clinicopathological features as well as response to radiotherapy in 100 cases of non-small cell lung cancer.

Clinicopathological	*N*	Positive	Negative	χ2	*P*
factors					
Age (years)					
<61	38	20	18	0.477	0.537
≥61	62	37	25		
Histopathology					
Squamous cell carcinoma	41	22	19	0.317	0.682
Adenocarcinoma	59	35	24		
Sex					
Male	80	46	34	0.410	1.000
Female	20	11	9		
TNM classification					
I + II	20	10	10	0.500	0.615
III + IV	80	47	33		
Response to IR (4 weeks)					
CR/PR	43	18	25	7.055	0.014
PD/SD	57	39	18		
Response to IR (12 weeks)					
CR/PR	46	20	26	6.355	0.015
PD/SD	54	37	17		

## Discussion

**Fig. 7:**
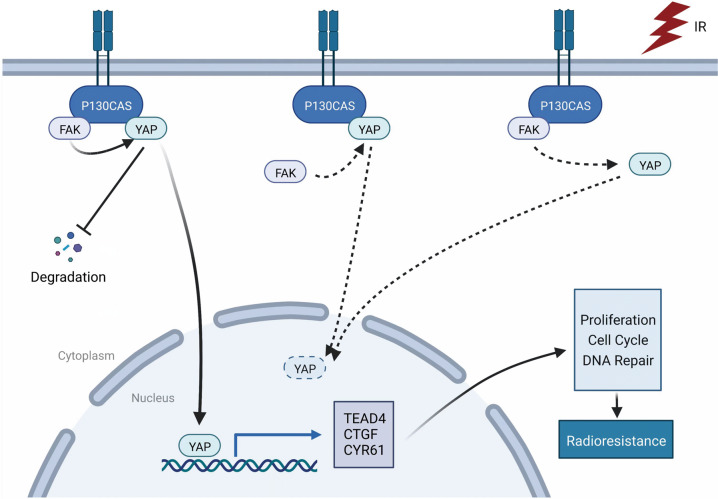
Pathway diagram for P130cas functional activity interaction with FAK and YAP after irradiation in lung cancer cells.

Our findings indicate that P130cas induces the activation of YAP, which results in the resistance of lung cancer cells to radiation, thus confirming the involvement of P130cas in the regulation of tumor radiosensitivity. We also demonstrate that P130cas, FAK, and YAP form a complex and that P130cas-FAK interaction is essential for facilitating the nuclear translocation of YAP in NSCLC (Fig. 7).

In the present study, we found that P130cas overexpression increased the proliferation of NSCLC cells, whereas P130cas deficiency inhibited the proliferation of NSCLC cells. The P130cas adaptor protein, also known as BCAR1, is one of the Src substrates co-localizes with paxillin in FAs and is involved in cell migration, survival, transformation, invasion, and cancer [17, 40]. P130cas is not an enzymatic protein but a docking and scaffolding protein that can mediate protein-protein interactions or directly interact with other proteins through its various domains and motifs. Like FAK, P130cas localizes to FAs and is phosphorylated by tyrosine kinases in response to adhesion signals [41]. In addition to its signaling role in FAs, P130cas also plays an essential role in regulating cell proliferation and survival in cancer cells [17, 40]. Silencing P130cas has been reported to reduce cell proliferation in breast cancer and ovarian carcinoma [42, 43]. However, proliferation was not affected by reducing P130cas expression in oral carcinoma [44]. Moreover, several studies concluded that P130cas was a downstream mediator of FAK only involved in mediating cell invasion and migration but not in cell growth [20]. Collectively, these findings suggested that the impaction of P130cas on cell proliferation might be in a tumor-specific context. Our previous study identified that overexpression of P130cas correlated with adverse clinicopathological parameters and was an independent prognostic factor for NSCLC patients’ survival [24]. The present results were consistent with the previous studies and indicated that P130cas could regulate cell proliferation in NSCLC.

Our study shows that the overexpression of P130cas downregulates radiosensitivity of lung cancer cells in vitro and in vivo and decreases the levels of cleaved PARP, cleaved caspase-3 and γ-H2AX induced by ionizing irradiation, which indicates the inhibition of apoptosis and DNA damage. The relationship between P130cas overexpression and radiosensitivity is undocumented to the authors’ knowledge. In a previous study, Beinke et al. [25] showed that both the protein expression and phosphorylation of P130cas were upregulated after treatment with a single radiation dose for 30 min. Our study extended the duration of this experiment and found a time-dependent increase in P130cas expression, which, together with earlier results, suggested that P130cas may be implicated in regulating tumor radiosensitivity. The association between P130cas and radiosensitivity revealed in this study suggests a new therapeutic target for the treatment of NSCLC patients.

Evidence over recent years indicates that YAP promotes resistance of tumor cells to various anti-cancer therapeutic methods, including radiation [30–34]. As a transcriptional effector, YAP is tightly regulated by upstream signaling molecules, among which the components of focal adhesions, such as protein kinases and adaptors, are considered pivotal mediators of cellular responses to external stimuli [16, 45]. P130cas is a vital adaptor protein involved in focal adhesion [17, 18, 40], but there has been no direct proof that P130cas regulates YAP activation, although Kaneko et al. reported that the impairment of P130cas activation was accompanied by the inhibition of YAP nuclear translocation [46]. In the present study, functional enrichment analysis of the DEGs based on the KEGG database showed that, compared with the control group, extracellular matrix (ECM)-receptor interaction and focal adhesion signaling pathways were significantly enriched in the P130cas-overexpressing group. Moreover, our results show that P130cas directly interacts with YAP, thereby promoting its stabilization, activation, and nuclear translocation. When YAP expression is inhibited, radiosensitivity is enhanced in lung cancer cells overexpressing P130cas both in vitro and in vivo. Our results show that P130cas may be an upstream regulator of YAP, which mediates tumor radiosensitivity through YAP activation and nuclear translocation, indicating the inhibition of the P130cas-YAP axis may present a promising novel target to increase the radiosensitivity of NSCLC.

FAK is the principal member of the focal adhesion complex, which binds to the SH3 domain of P130cas and facilitates the phosphorylation of P130cas to activate downstream signaling pathways [15, 47]. A previous study demonstrated that the inhibition of FAK activity blocked YAP nuclear accumulation [37]. A previous study also revealed that overexpression of FAK resulted in increased tyrosine phosphorylation of P130cas [48]. We thereby overexpressed FAK and observed that the phosphorylated P130cas (data not shown) and nuclear YAP levels were increased while the level of P130cas protein did not show visible changes. However, knockdown of P130cas significantly decreased nuclear YAP expression induced by FAK overexpression. Our data suggested that FAK may promote YAP activation by inducing phosphorylation of P130cas. In the same study, the authors also revealed that co-expression of P130cas with FAK further increased cell migration [48]. Hence, we overexpressed P130cas in A549 cells and knockdown P130cas in H1299 cells. Surprisingly, western blotting results showed that the levels of both Tyr397 and Tyr925 phosphorylation of FAK were significantly elevated by P130cas overexpression and decreased by P130cas depletion. Subsequent results found that both FAK inhibitor and FAK-specific siRNA significantly attenuated increasing growth, reversed radioresistance and decreased YAP nuclear accumulation induced by P130cas overexpression. Our results suggested that overexpression of P130cas can also promote the phosphorylation of FAK, which facilitates cell proliferation, YAP activation, and radioresistance of NSCLC cells. Although many reports demonstrated that overexpression of P130cas was associated with adverse clinicopathological parameters, only a few researchers investigated the impaction of P130cas overexpression of FAK and suggested that overexpression of P130cas did not affect FAK [20–24]. Our data indicated that the effect of P130cas on regulating FAK might be in a tumor-specific context. Together with these studies, our current findings suggest that P130cas and FAK may cooperate to promote YAP’s activation and nuclear accumulation, thereby decreasing the radiosensitivity of NSCLC cells.

The association between P130cas and FAK is crucial for the integrity and function of focal adhesions [17, 18, 40]. The phosphorylation of P130cas depended on its binding activity since it was abolished by the mutation of the binding site on FAK [48]. The proline-rich region of FAK spanning amino acids 712–718 has been mapped as a binding site for the SH3 domain of P130cas [38]. Interestingly, YAP also has a proline-rich region that is predicted to bind with signaling molecules containing SH3 domains, and a WW domain is predicted to bind with signaling molecules that contain a proline-rich region [39]. As a result, we performed immunoprecipitation to test the interactions among P130cas, FAK and YAP and confirmed that P130cas could form a triple complex with FAK and YAP. Since the SH3-domain of P130cas is the putative binding site for FAK and YAP, SH3-deficient P130cas mutant lentivirus delivering effectively blocks the bindings between P130cas and FAK or P130cas and YAP. Similarly, the proline-rich region (AA 712–718) of FAK is the putative binding site for P130cas and YAP, transfected with P712/715A FAK mutant also effectively block the bindings between FAK and P130cas or FAK and YAP. As YAP’s proline-rich region (SH3 binding motif) is the putative binding site for P130cas, the following co-IP results showed that transfected with SH3-binding motif deficient YAP mutant blocks the bindings between YAP and P130cas. Beyond that, we surprisingly found that blocking the binding between YAP and P130cas also prevented the interaction between YAP and FAK. Next, we abolished the putative FAK binding site of YAP (ΔWW) to block the interaction between YAP and FAK. Intriguingly, we observed that transfected with the WW-deficient YAP mutant could not effectively block either YAP-FAK interaction or YAP-P130cas interaction. Together, the above results provide hints about the P130cas-FAK-YAP complex that both FAK and YAP could directly interact with P130cas, whereas FAK-YAP might indirectly interact with each other and P130cas was essential for the interaction between FAK and YAP. In an effort to clarify this, we further evaluated the impact of the integrity of the P130cas-FAK-YAP complex on YAP activation and radiosensitivity. Our results suggested that both P130cas-ΔSH3 mutant and FAK-712/715A mutant prominently reduced the nuclear accumulation and activation of YAP, as well as the increasing radioresistance caused by P130cas or FAK overexpression. Therefore, targeting P130cas-FAK interaction may be more cost-effective to overcome the YAP activation mediated radioresistance in NSCLC.

Using the data from the public database and our clinical samples, we further evaluated the clinical significance of P130cas. As we reported in the previous study, P130cas was associated with adverse clinical outcomes in NSCLC patients [24]. Moreover, our study has confirmed that the expression of P130cas correlated with YAP expression and indicated poor ORR in NSCLC patients who underwent radiation therapy. To the best of our knowledge, there are no reports available that address the relationship between P130cas and YAP in literature and the effect of P130cas on response to radiation therapy. Our findings shed new light on the associations between P130cas and YAP. Furthermore, the present study may provide an alternative strategy to overcome P130cas-YAP axis-induced radioresistance in NSCLC. Previous studies have demonstrated that ECM-receptor interaction regulated YAP activation and nuclear translocation through small GTPase in diverse biological processes. CDC42 has been proven to be an essential regulator of YAP during kidney development [49]. RhoA GTPase was previously reported as a positive upstream regulator of YAP activity via its inhibition of LATS1/2 kinases] [50]. ROCK-Rho GTPases are known to sustain transcriptional activation of both TEAD and SRF through their ability to promote actin polymerization and thus potentiate cytoskeletal tension and nuclear shuttling of YAP/TAZ and MRTF-A [51]. Interestingly, our unpublished data showed that, after binding with YAP, P130cas might recruit and activate Rac1 to drive the nuclear translocation of YAP (Data not shown). However, the specific regulatory mechanism is still unclear and needs to be further studied.

In conclusion, we used in vitro and in vivo models to characterize the relationship and interaction of the P130cas-FAK-YAP axis and investigated the role of P130cas in regulating the radiosensitivity of NSCLC cells. In doing so, the present study provides several lines of evidence that P130cas cooperate with FAK to promote the nuclear accumulation and activation of YAP, thereby inducing radioresistance in NSCLC. First, silencing P130cas abolished the ability of FAK to activate YAP while inhibiting FAK also capable of reducing the activation of YAP caused by P130cas overexpression. Second, in the regulation of YAP activity in NSCLC cells, beyond phosphorylation of P130cas, the overexpression of P130cas matters. These results strongly suggested that focusing on the overexpression of P130cas may give us a new insight into overcoming the YAP-mediated radioresistance in NSCLC patients. Finally, blocking the binding between P130cas and FAK may be an effective therapeutic target to reduce the YAP-mediated radioresistance in NSCLC.

## Supplementary information

Supplementary Figures

Checklist

Original Data File

## Data Availability

The datasets used and/or analyzed during the current study are available from the corresponding author on reasonable request.
