# Supplementary material for: P130cas-FAK interaction is essential for YAP-mediated radioresistance of non-small cell lung cancer
Source: Cell Death Dis. 2022 Sep 10;13(9):783. doi: 10.1038/s41419-022-05224-7 (PMC9464229; doi:10.1038/s41419-022-05224-7)
Supplement: Supplementary file 1 — Supplementary Figures [file 41419_2022_5224_MOESM1_ESM.doc]

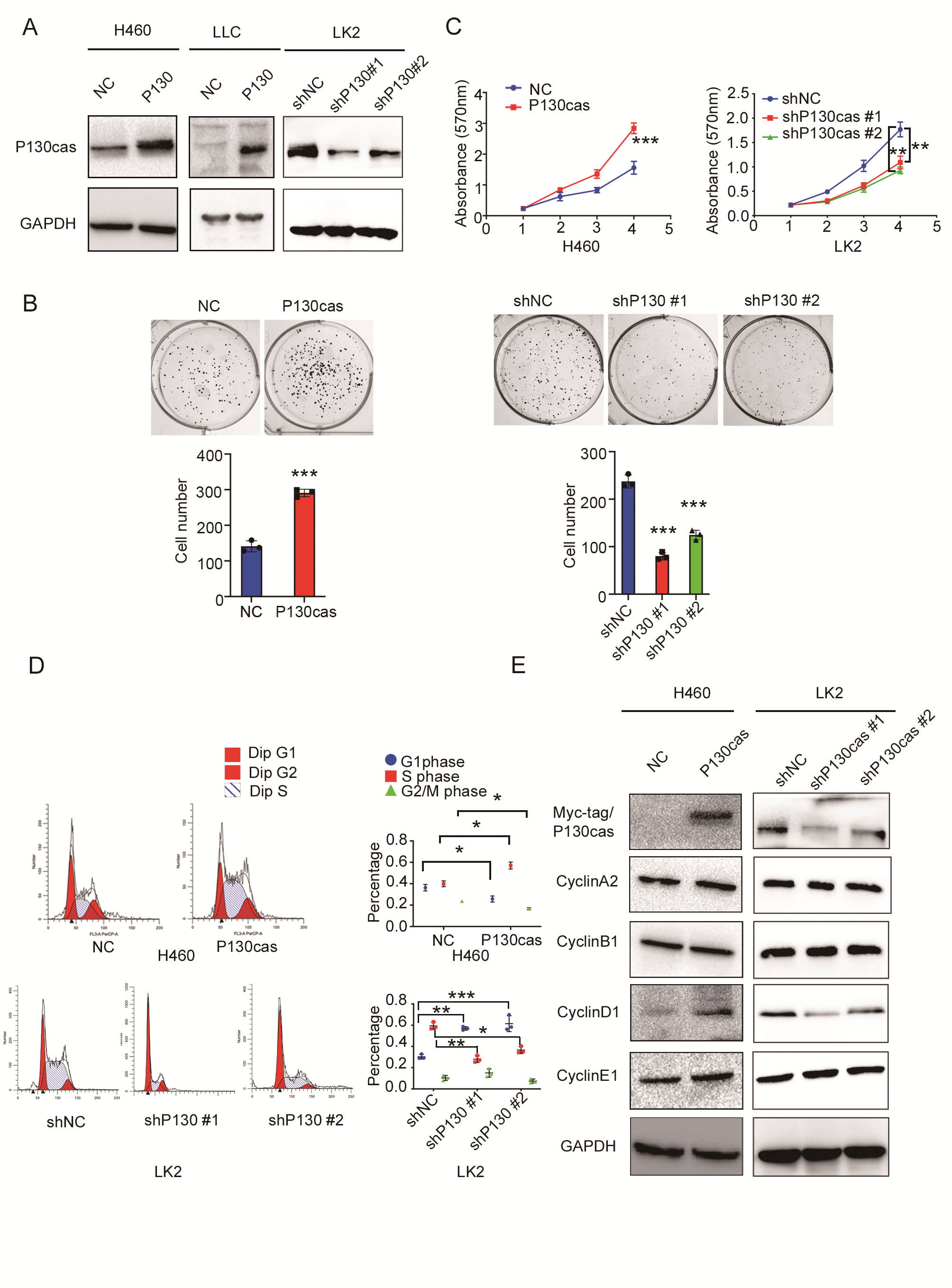


**Supplementary Figure 1. Overexpression of P130cas promotes NSCLC proliferation**

(A) Stable up- or downregulation of P130cas expression was confirmed by western blotting in H460 and LLC or LK2 cells, respectively (B) Colony formation assay (B), MTT assay (C) and cell cycle analysis (D) using the H460-NC and -P130cas clones, or LK2-NC and –shP130cas #1 and #2 clones Quantification data are expressed as average±SD of three independent experiments (t-test, two-sided, *P<0.05, ** P<0.01, ***P<0.001). (E) Western blotting assay detected the expression of cell-cycle related proteins in the H460-NC and -P130cas clones and LK2-NC and –shP130cas #1 and #2 clones. Each carried out in triplicate. For Western blot experiments, the samples derive from the same experiment and the gels/blots were processed in parallel.

**
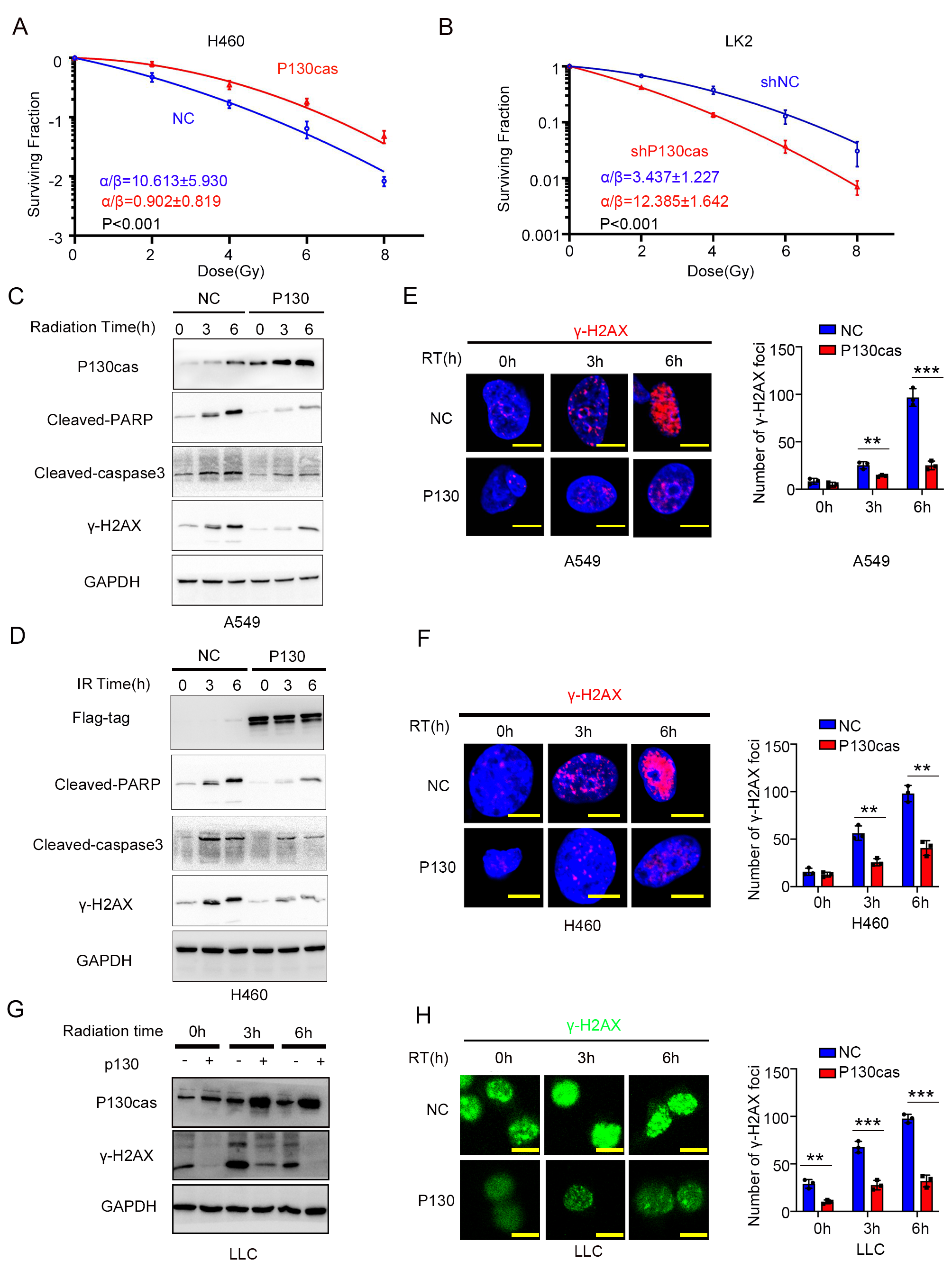
Supplementary Figure 2. Overexpression of P130cas accelerated radioresistance**

(A) Clonogenic cell survival fraction curves fitted with the linear-quadratic model with or without overexpressing P130cas in H460 cells. (B) Clonogenic cell survival fraction curves fitted with the linear-quadratic model with or without silencing P130cas in LK2 cells. (C and D) Immunoblotting was used to evaluate the expression of cleaved-caspase-3, cleaved PARP and γ-H2AX. Immunofluorescence assay (E and F, scale bar=10μm) was used to evaluate the number of γ-H2AX foci at various time points after IR in the A549 or H460-NC and -P130cas clones. (G-H) Western blot and immunofluorescence assay illustrated the expression of γ-H2AX and the number of γ-H2AX foci in LLC cells with or without P130cas overexpression after radiation in different time course, scale bar: 10μm, each carried out in triplicate. For Western blot experiments, the samples derive from the same experiment and the gels/blots were processed in parallel.


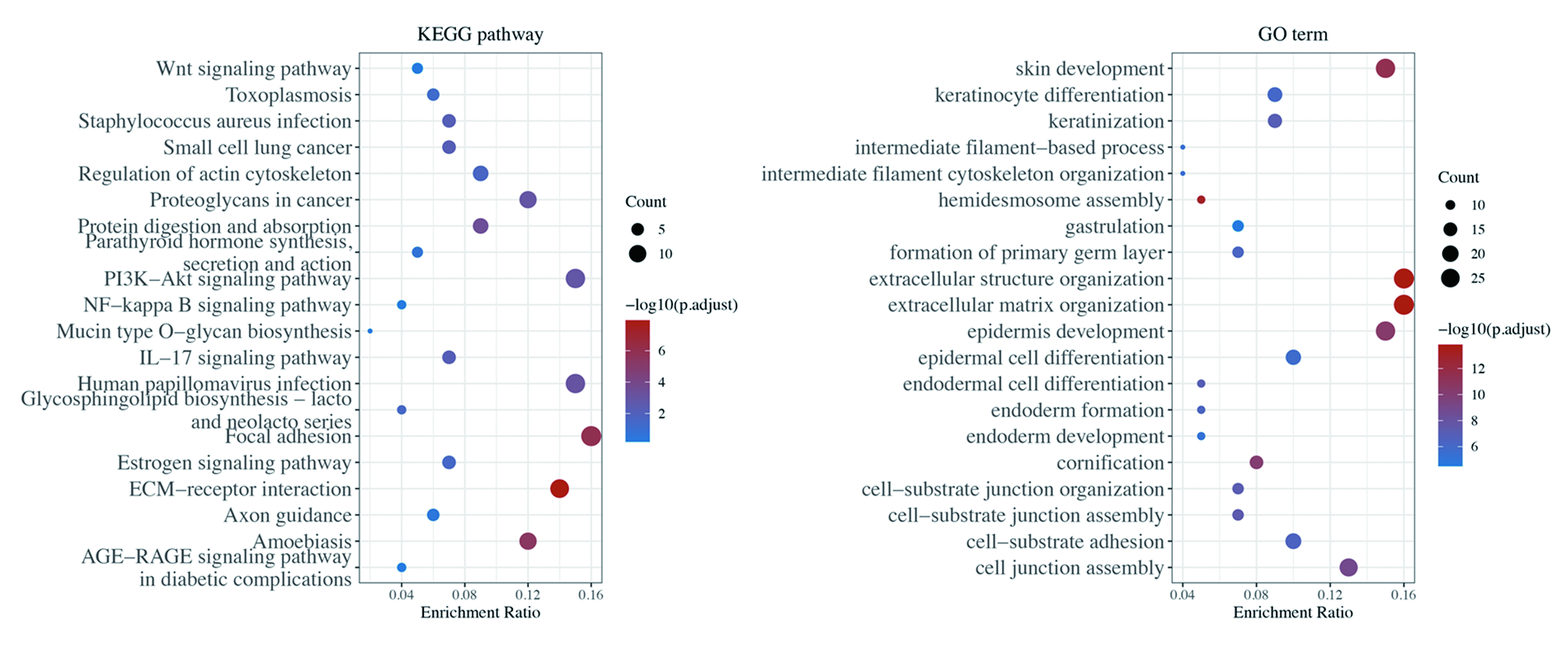


**Supplementary Figure 3. KEGG and GO term analysis for P130cas expression in NSCLC**

(A-B) KEGG and GO term analysis was performed to explore the signalling pathway positively enriched in NSCLC with high P130cas expression.


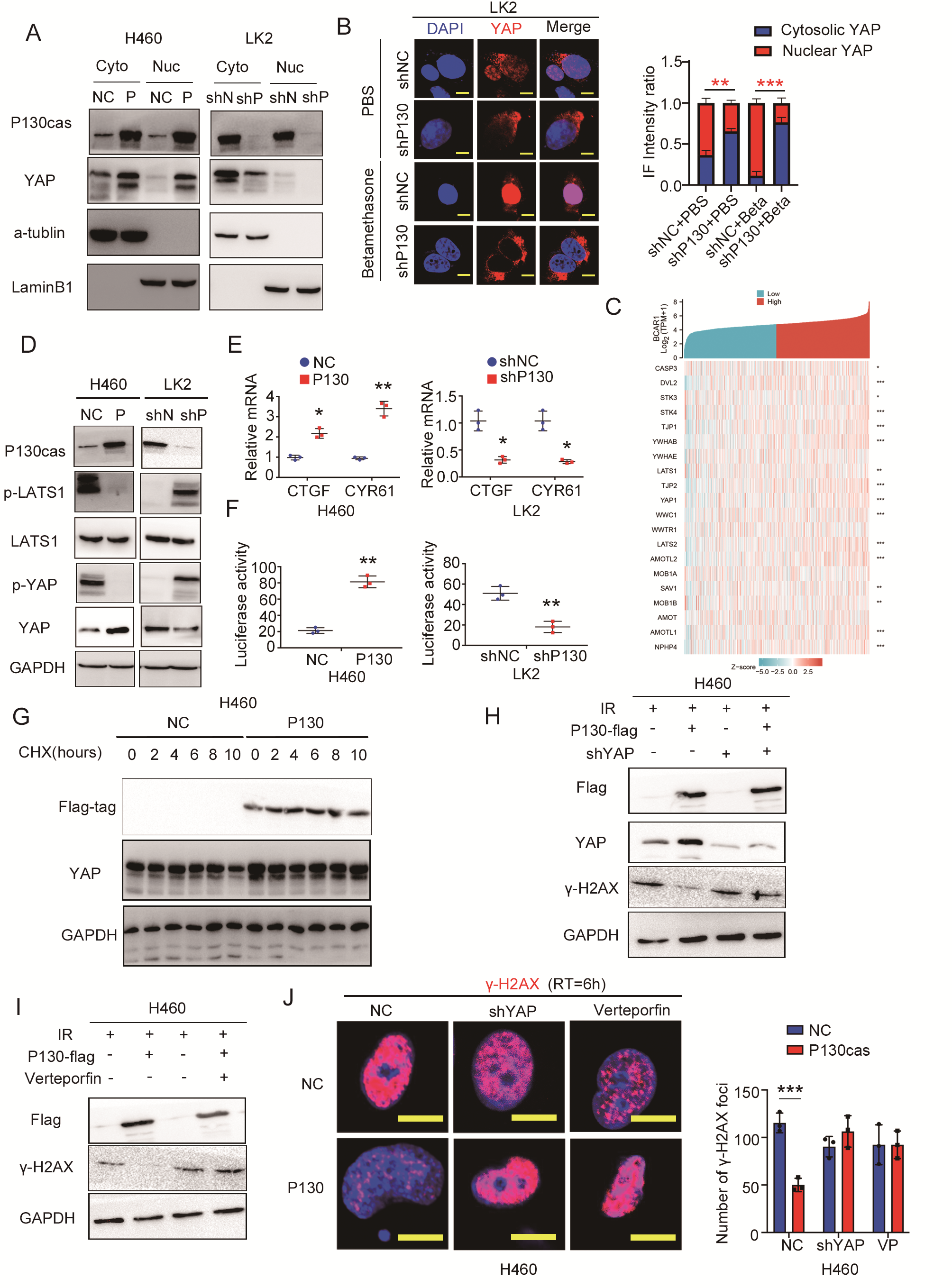


**Supplementary Figure 4. Overexpression of P130cas promoted radioresistance by stabilizing YAP**

In the H460-NC and -P130cas clones, and LK2-NC and –shP130cas clones, immunoblotting (A) were used to detect the nuclear expression and distribution of YAP (α-tubulin or LaminB1 were served as cytosolic or nuclear protein internal control). In the LK2-NC and –shP130cas clones with or without betamethasone, immunofluorescence staining was used to evaluate the subcellular localization of YAP (B, scale bar=10μm) (C) GEPIA database analysis revealed the correlation between P130cas expression and Hippo signalling pathway components. In the H460-NC and -P130cas clones, and LK2-NC and –shP130cas clones, western blotting assay was used to detect the phosphorylation of LATS1 and YAP (D), qPCR assay was used to investigate the alteration of the target genes of YAP (E), luciferase reporter assay was used to identify binding activity between YAP and TEAD4 promoter (F). After being treated with CHX at indicated time point, the expression of YAP was evaluated by western blotting in the H460-NC and -P130cas clones (G). In the H460-NC and -P130cas clones irradiated by 5Gy X-ray, after incorporation with YAP-shRNA or verteporfin, western blotting (H-I) and immunofluorescence (J) were used to observe the number of γ-H2AX foci (scale bar=10μm). *P<0.05, ** P<0.01, ***P<0.001, each carried out in triplicate. For Western blot experiments, the samples derive from the same experiment and the gels/blots were processed in parallel.


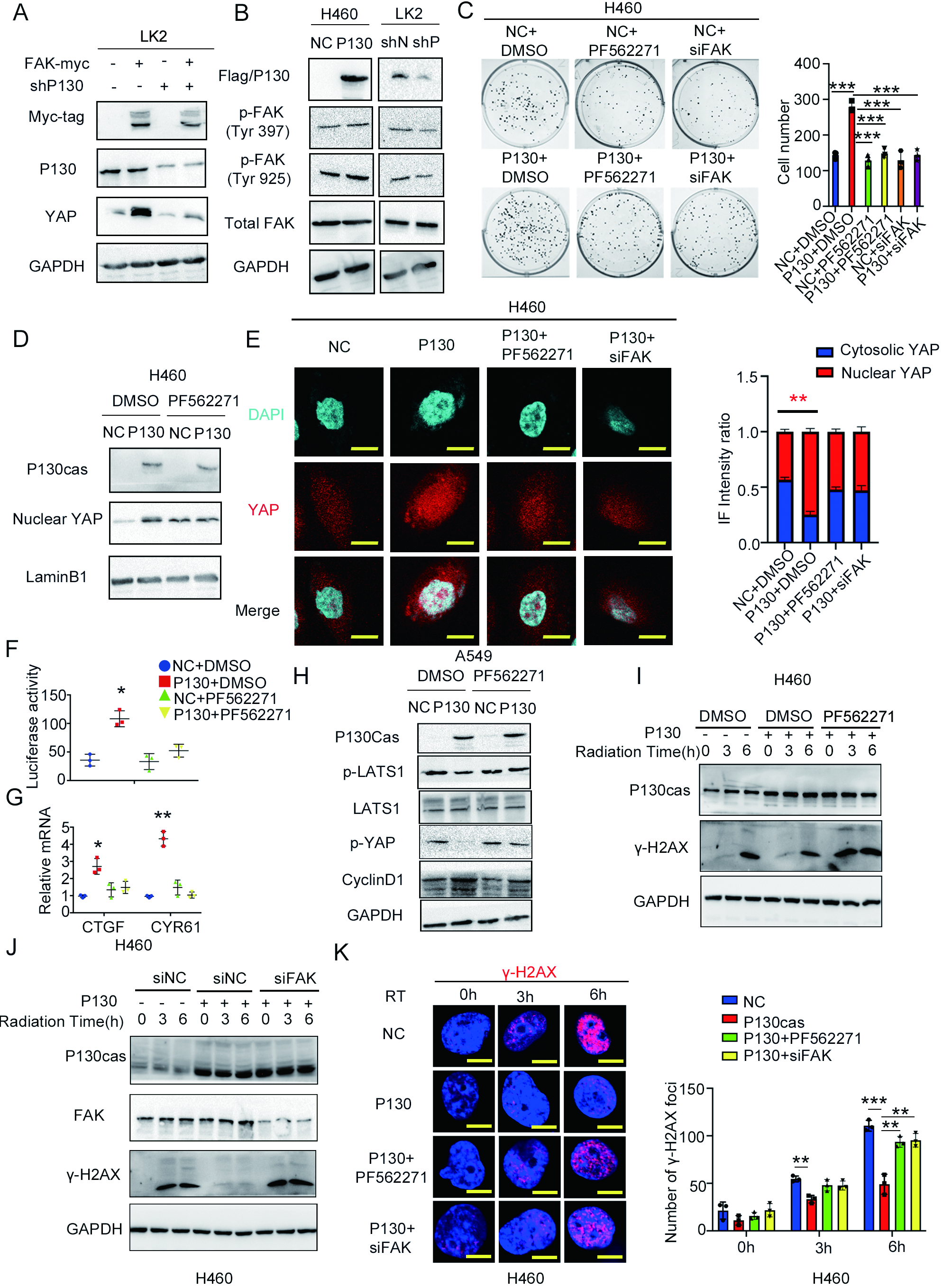


**Supplementary Figure 5. P130cas activated YAP and induced radioresistance via FAK signaling**

(A) Immunoblotting of Myc-tag, P130cas (P130), YAP and GAPDH in LK2 cells transfected with FAK-myc alone or in combination with P130cas shRNA. (B) Immunoblotting of Flag/P130cas (P130), FAK-Tyr397, FAK-Tyr925, total FAK and GAPDH in H460 cells overexpressing P130cas or in LK2 cells transfected with P130cas shRNA. (C) Representative images and data quantification of colony formation by H460 cells overexpressing P130cas alone versus in combination with PF562271 or FAK siRNA. *, P<0.05. (D) In H460 cells overexpressing P130cas alone or in combination with PF562271, after nucleoplasmic separation, using immunoblotting to evaluate P130cas, nuclear YAP and LaminB1, using immunofluorescence to evaluate YAP subcellular distribution (E), using luciferase reporter assay (F) and qPCR assay(G) to detect the downstream gene activity of YAP (*t* test. *, P<0.05, **, P<0.01). (H) In A549 cells overexpressing P130cas alone or in combination with PF562271, using immunoblotting to check phosphorylation of LATS1 and YAP as well as CyclinD1. In H460 cells overexpressing P130cas alone or in combination with PF562271/FAK-siRNA, immunoblotting of P130cas, γ-H2AX and GAPDH at the indicated time points after 5Gy ionizing radiation (I-J), representative immunofluorescence images of the number of γ-H2AX foci at the indicated time points after 5Gy ionizing radiation (K, scale bar=10μm). Each carried out in triplicate. For Western blot experiments, the samples derive from the same experiment and the gels/blots were processed in parallel.


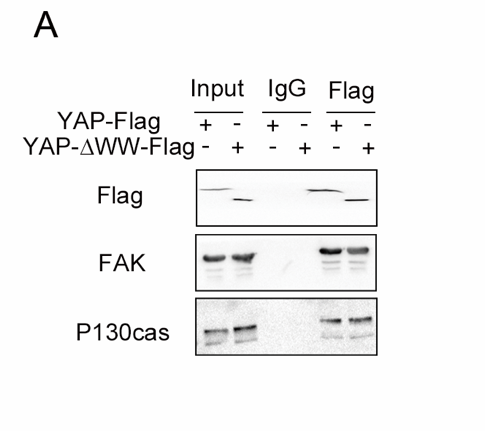


**Supplementary Figure WW domain is dispensable for P130cas, FAK and YAP triple complex**

(A) YAP was immunoprecipitated by Flag antibody from A549 cells transfected with Flag-YAP or Flag-YAP-ΔWW and immunoblotted with indicated antibodies. Each carried out in triplicate. For Western blot experiments, the samples derive from the same experiment and the gels/blots were processed in parallel.
