# Supplementary material for: P130cas-FAK interaction is essential for YAP-mediated radioresistance of non-small cell lung cancer
Source: Cell Death Dis. 2022 Sep 10;13(9):783. doi: 10.1038/s41419-022-05224-7 (PMC9464229; doi:10.1038/s41419-022-05224-7)

Figure 1


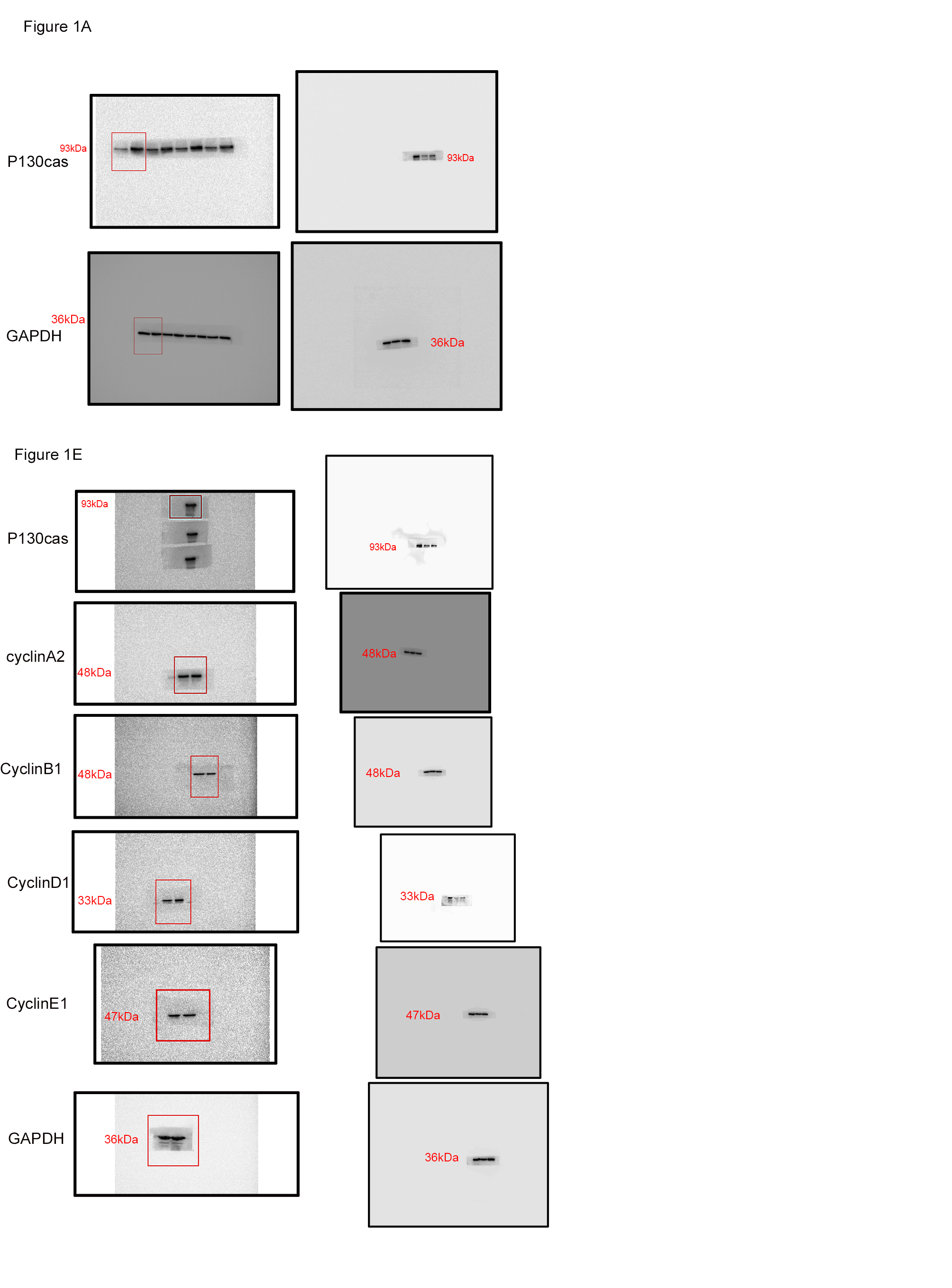


Figure 2


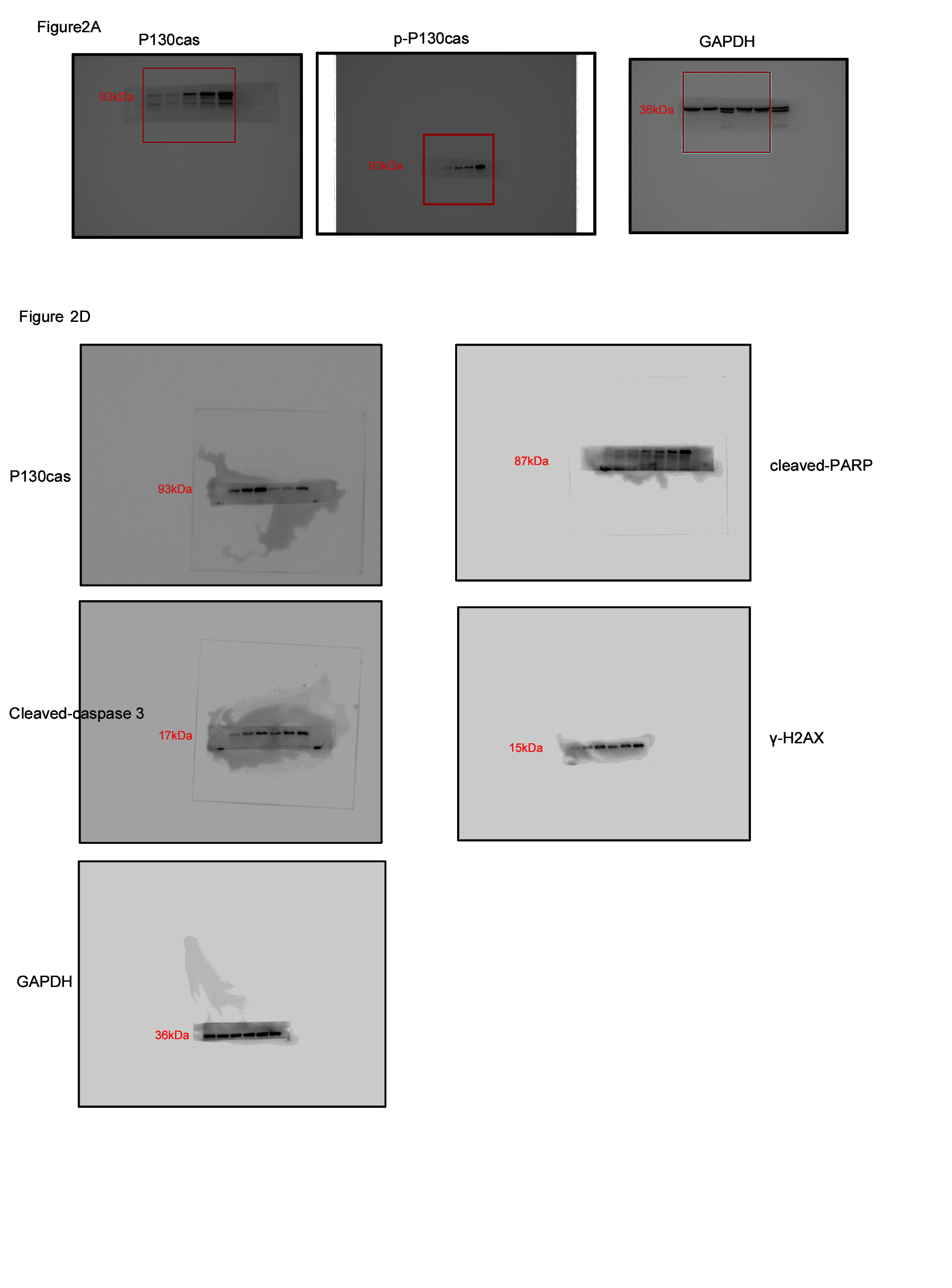


Figure 3B-D


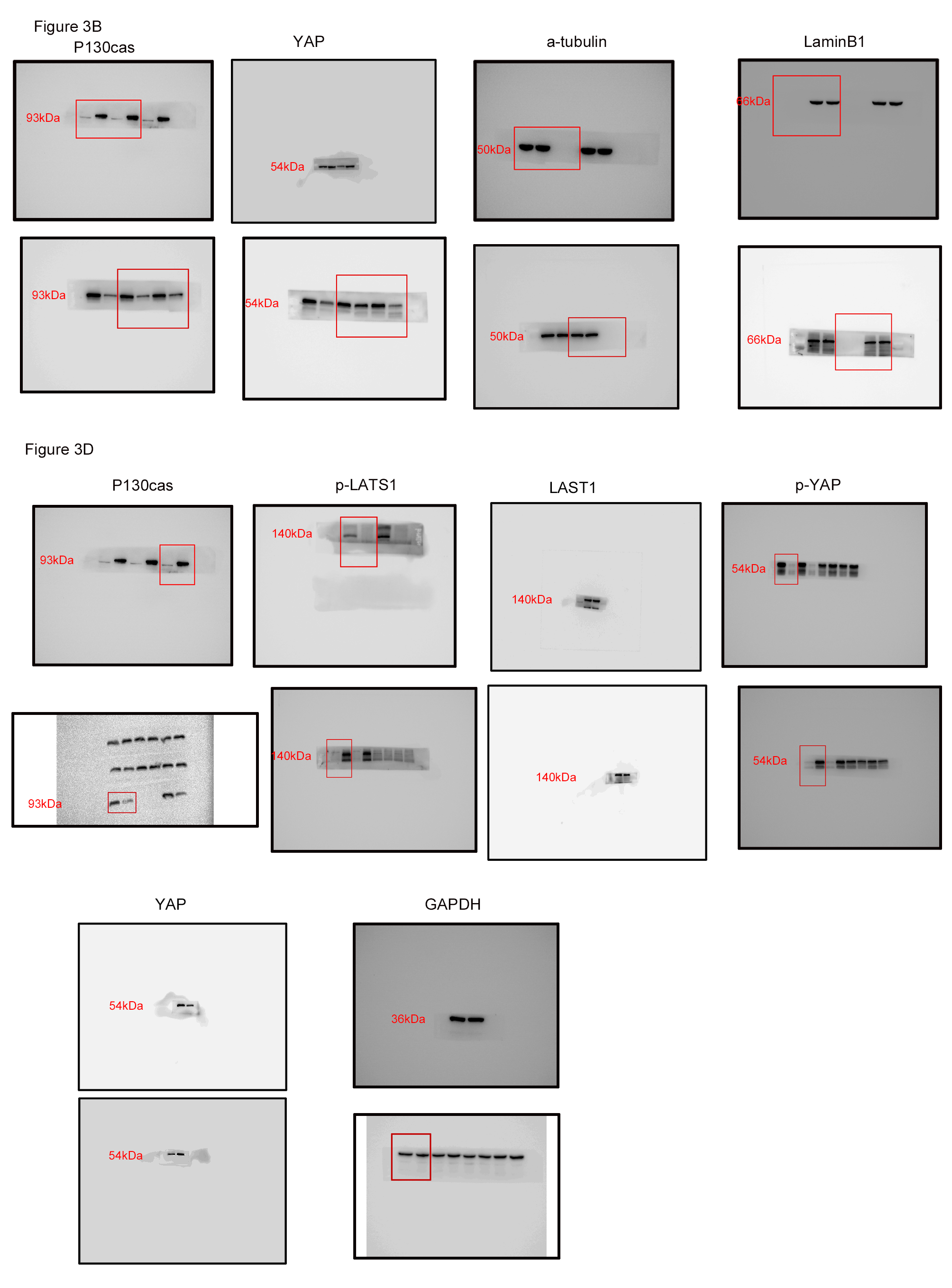


Figure 3G-H-I


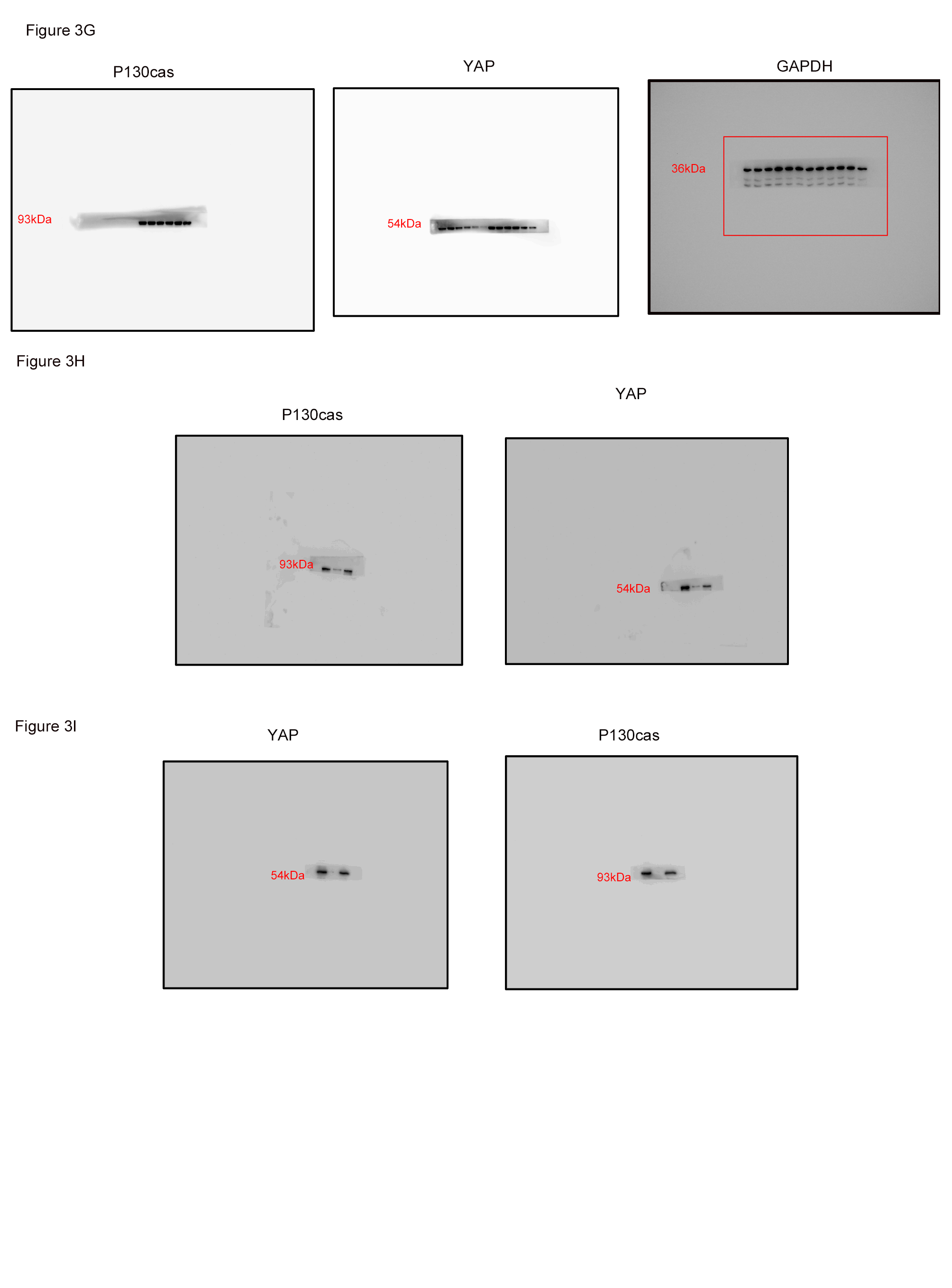


Figure 3K-L


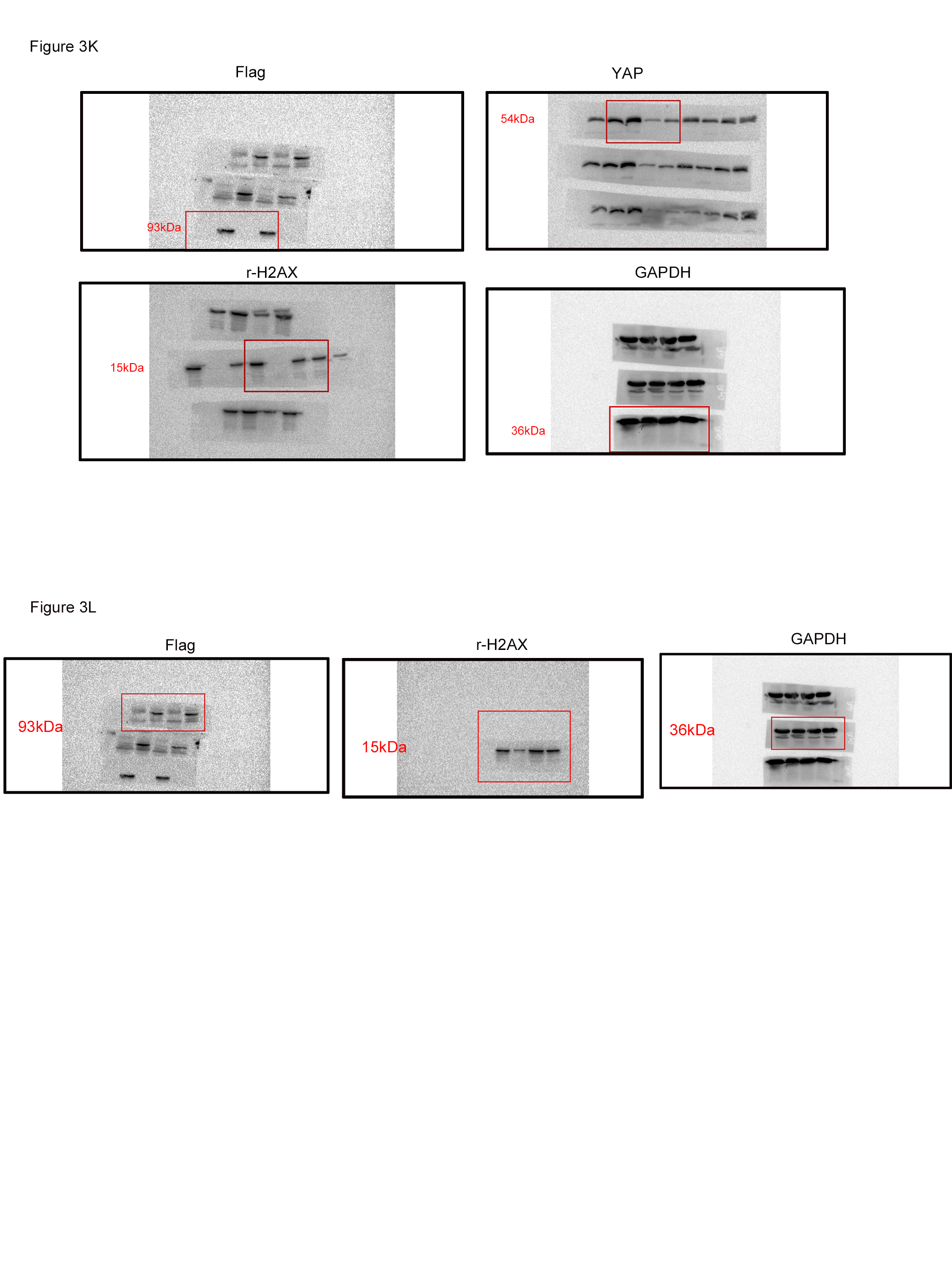


Figure 4A-B


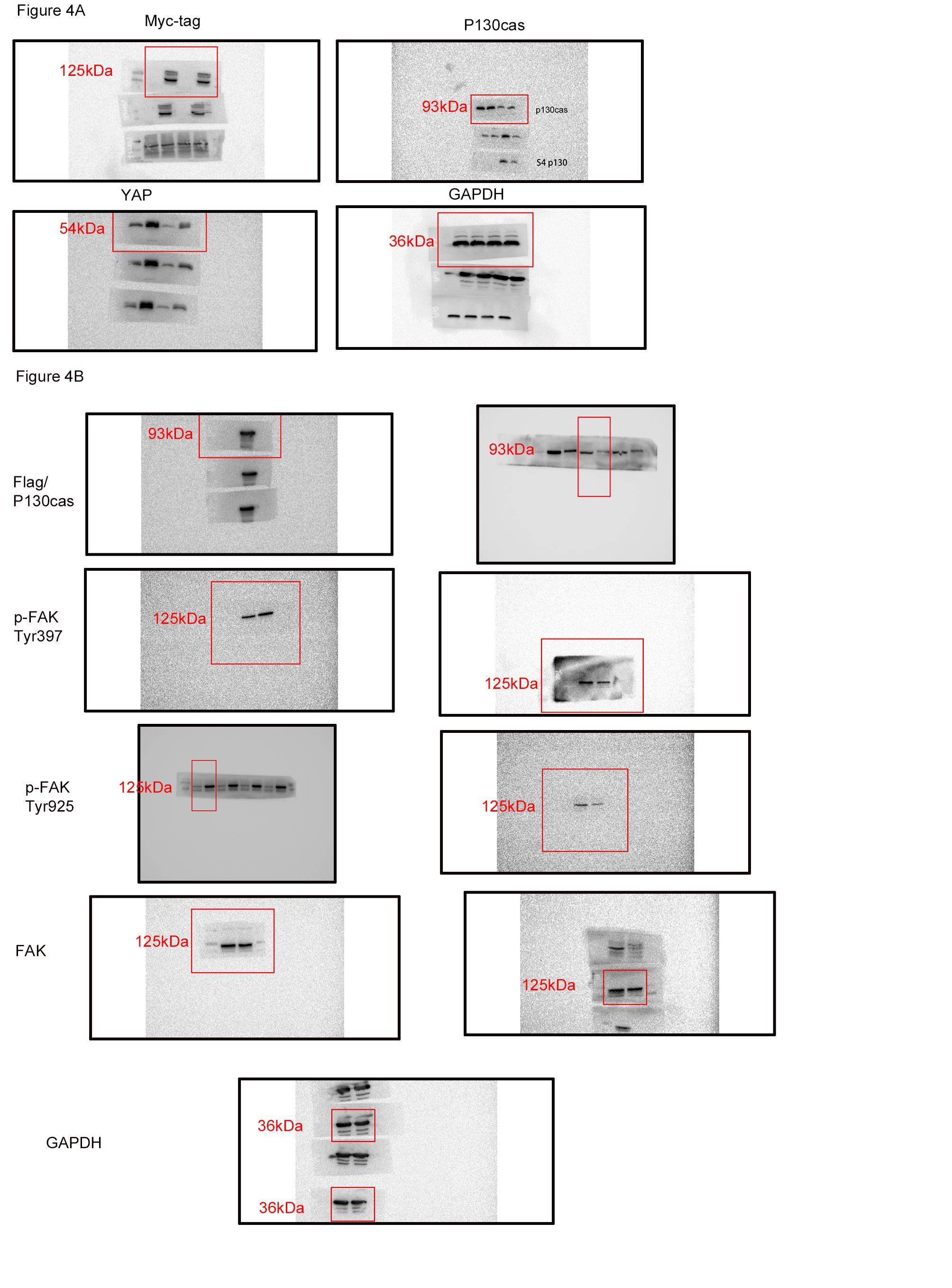


Figure 4D-G-H


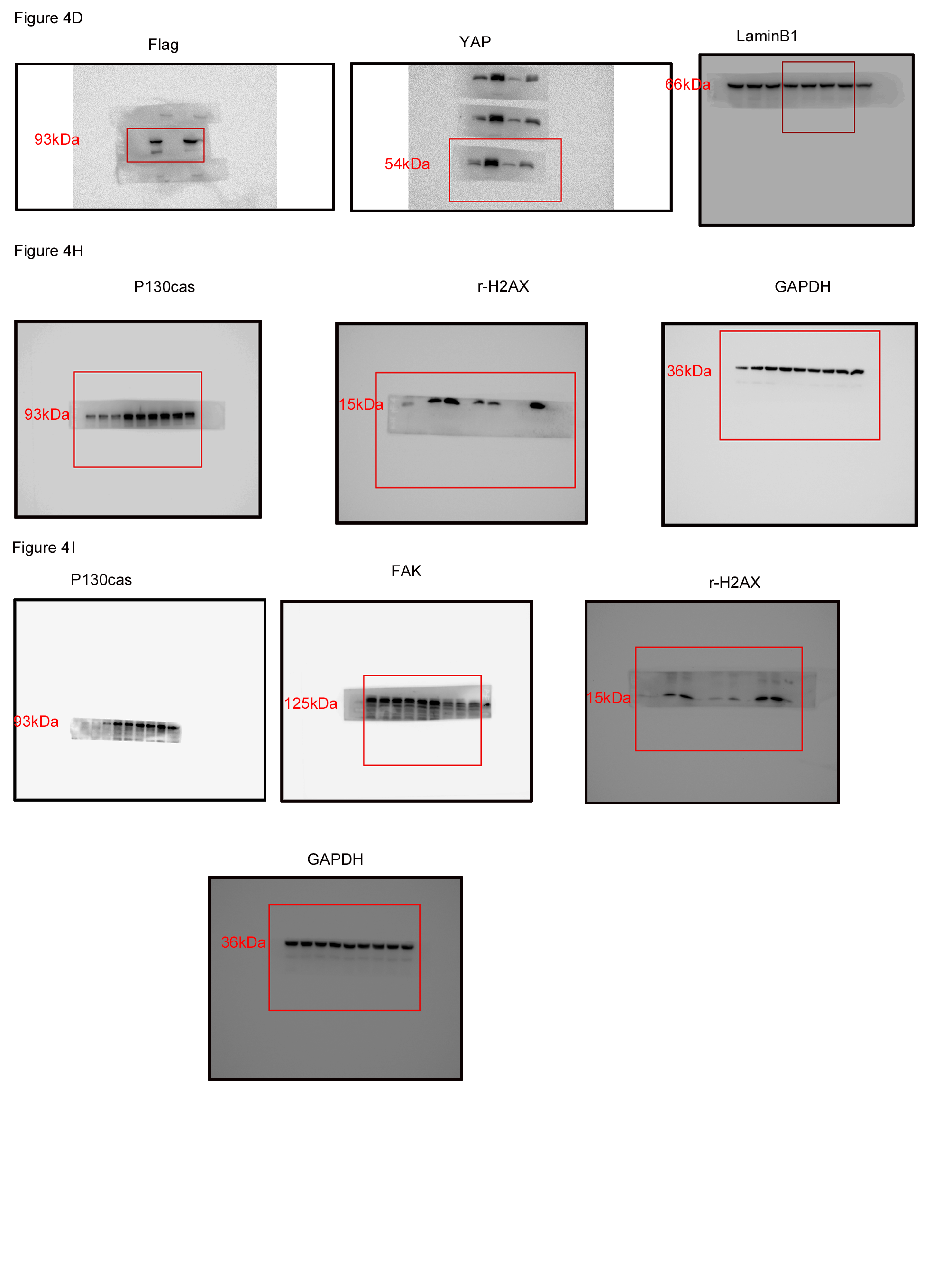


Figure 5A-D


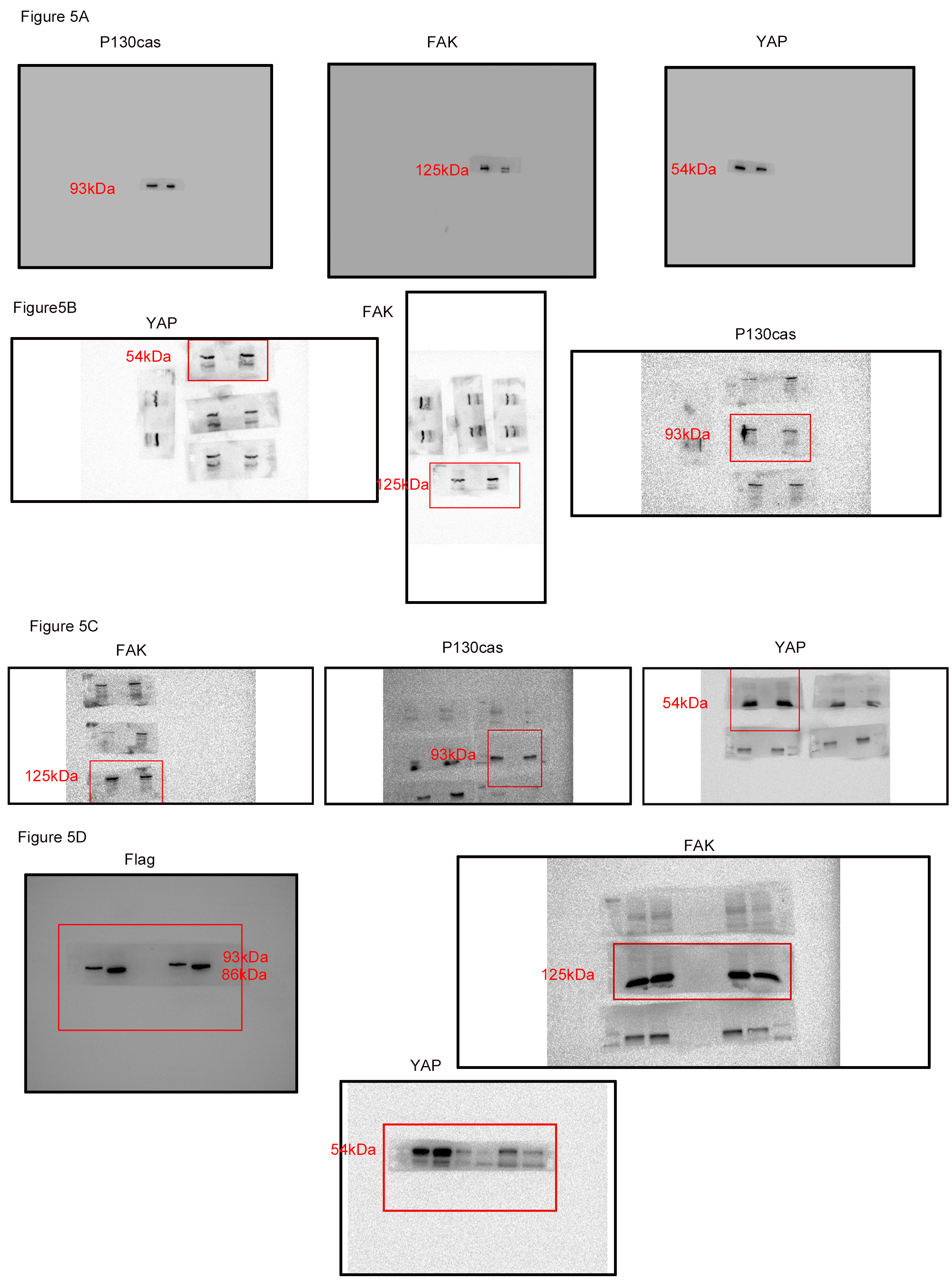


Figure 5E-G


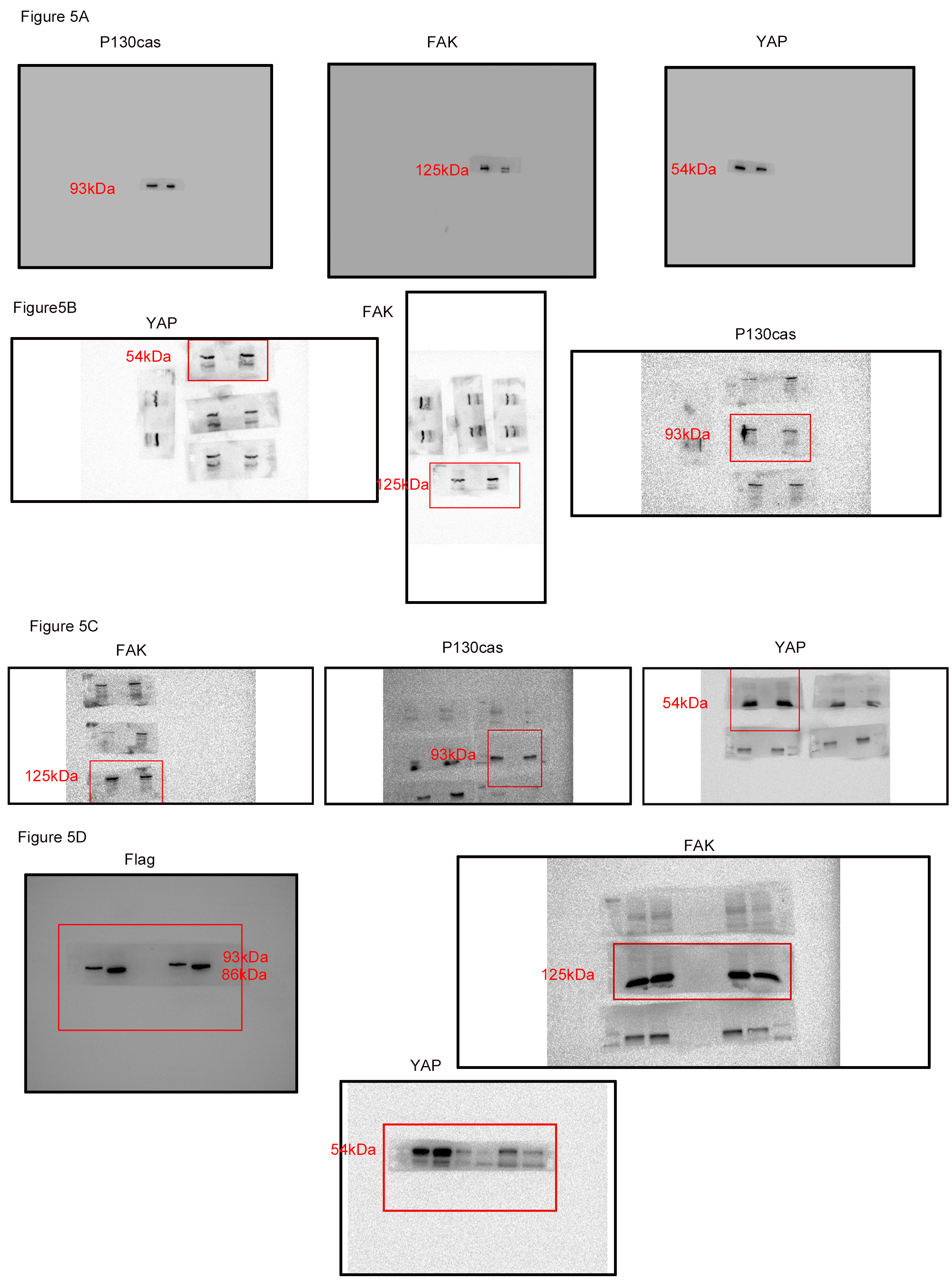


Figure 5N-O


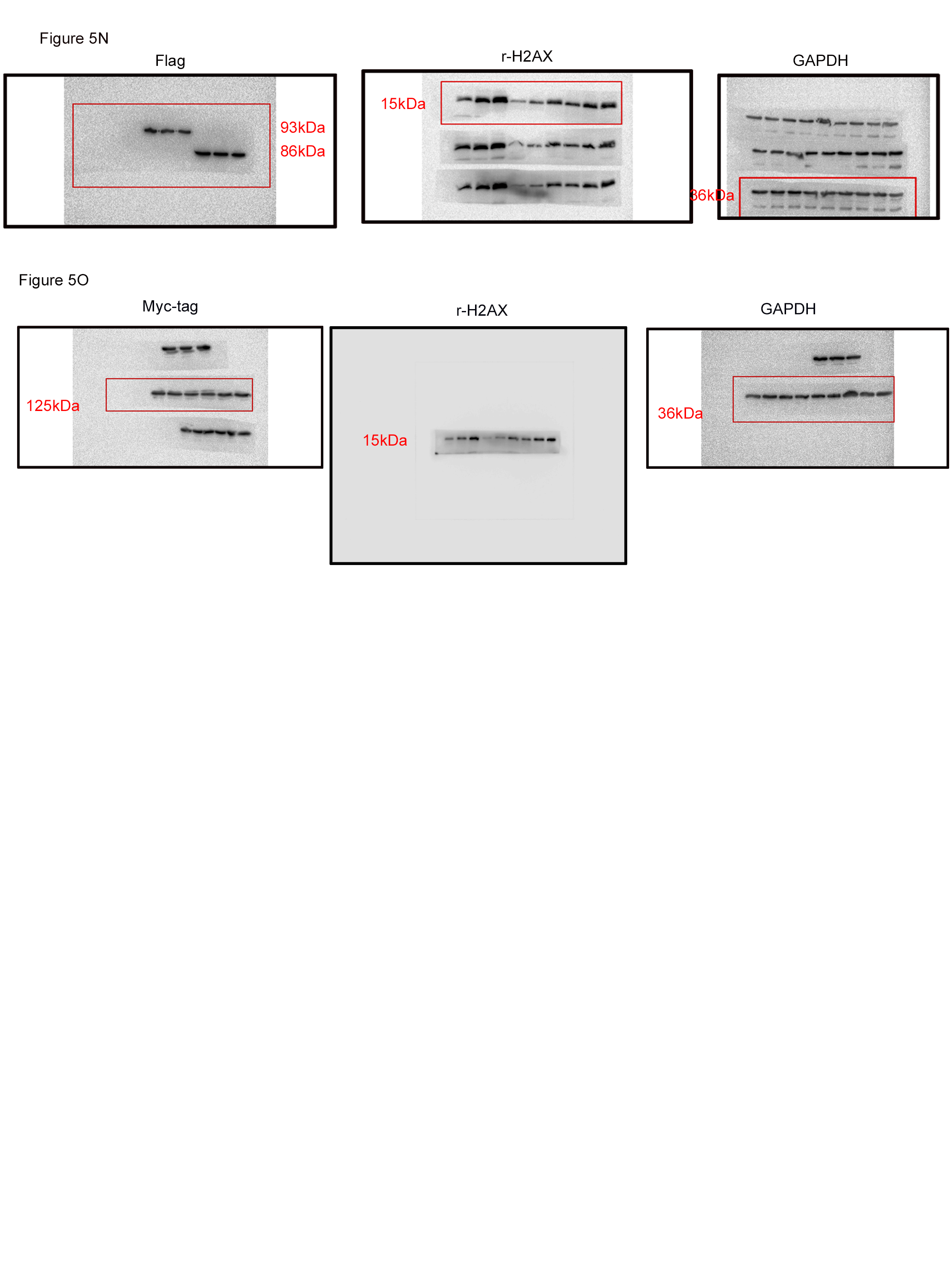


Supplementary Figure 1


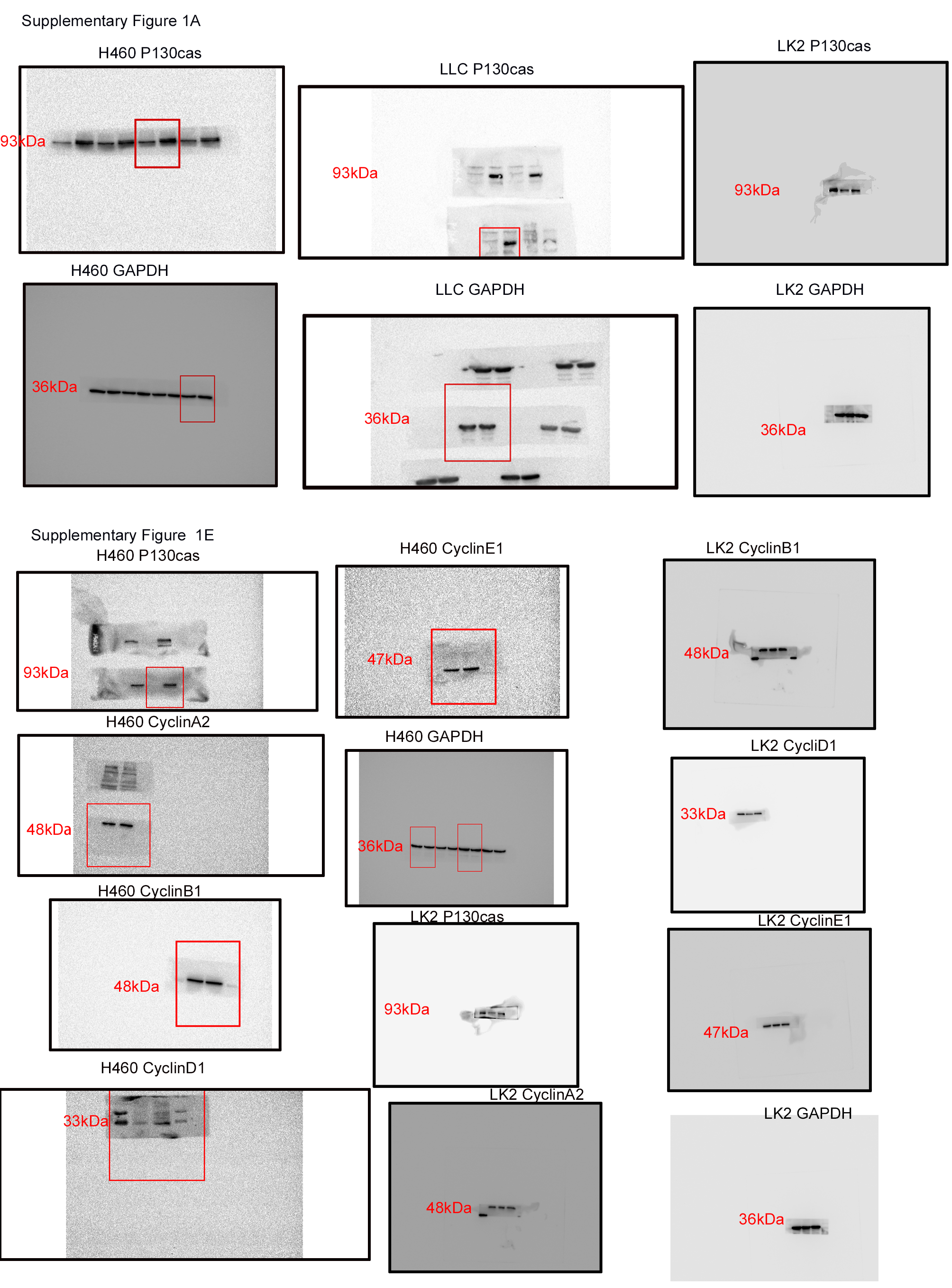


Supplementary Figure 2C-D


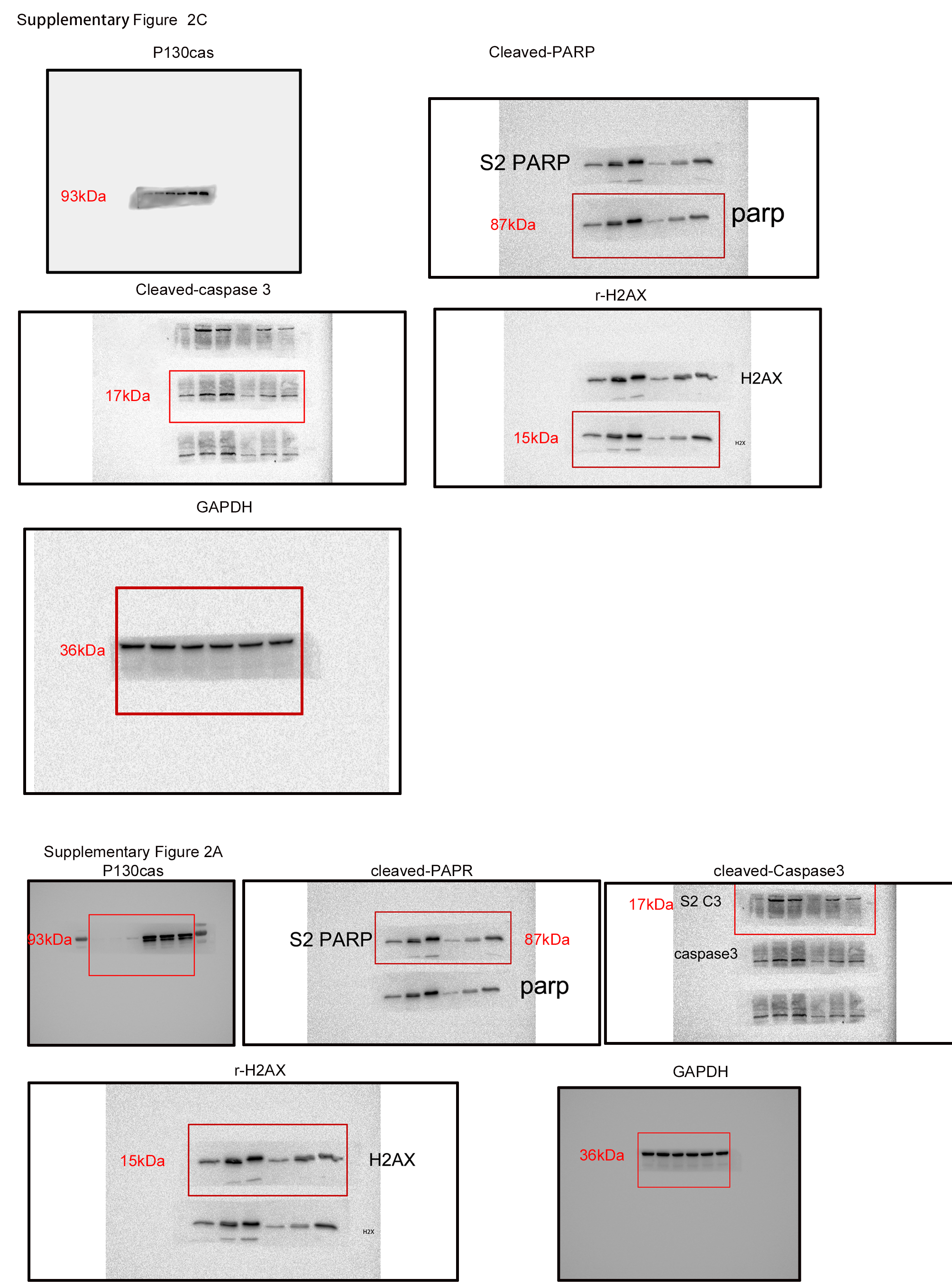


Supplementary Figure 2G


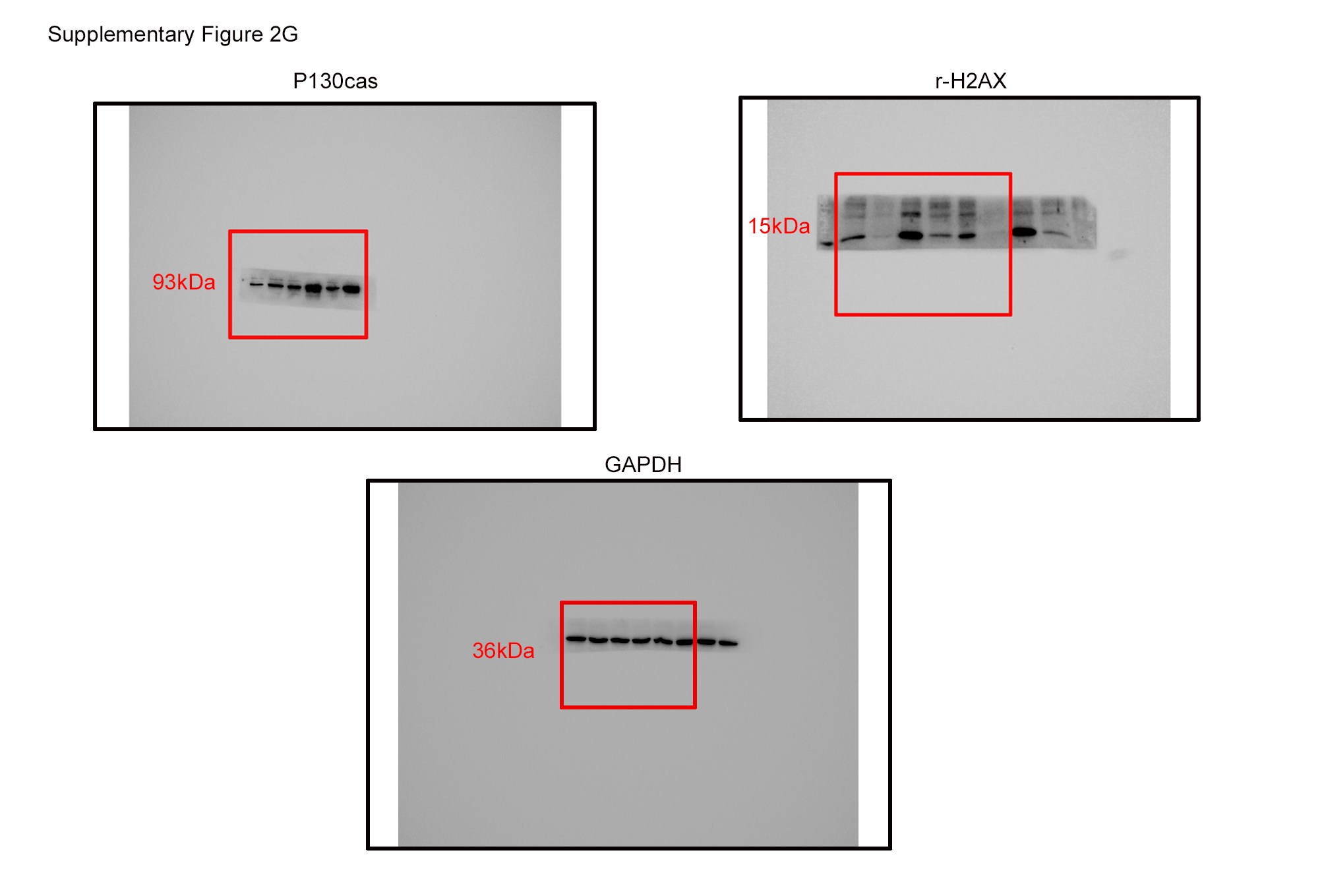


Supplementary Figure 4A-D-G


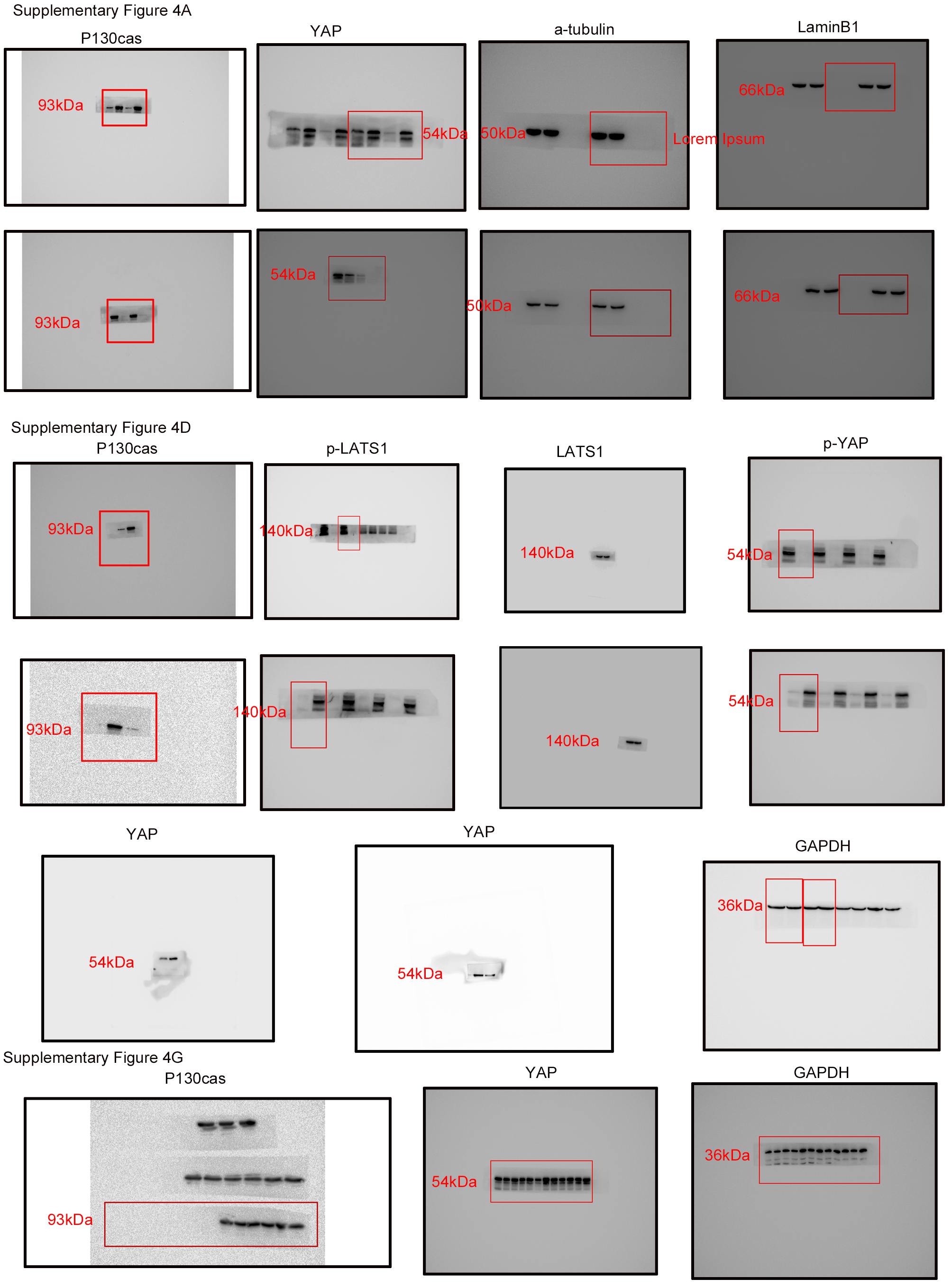


Supplementary Figure 4H-I


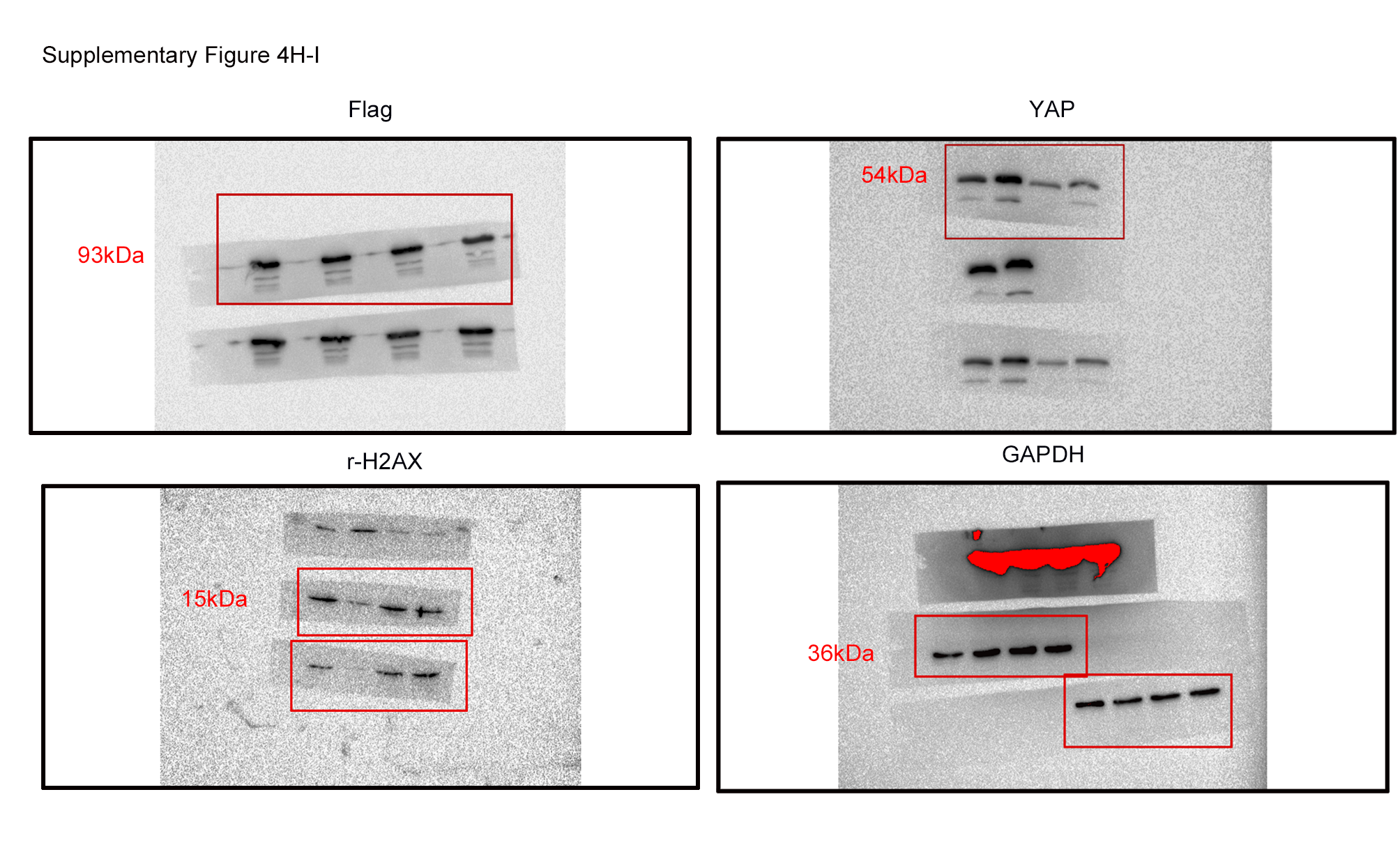


Supplementary Figure 5A-B


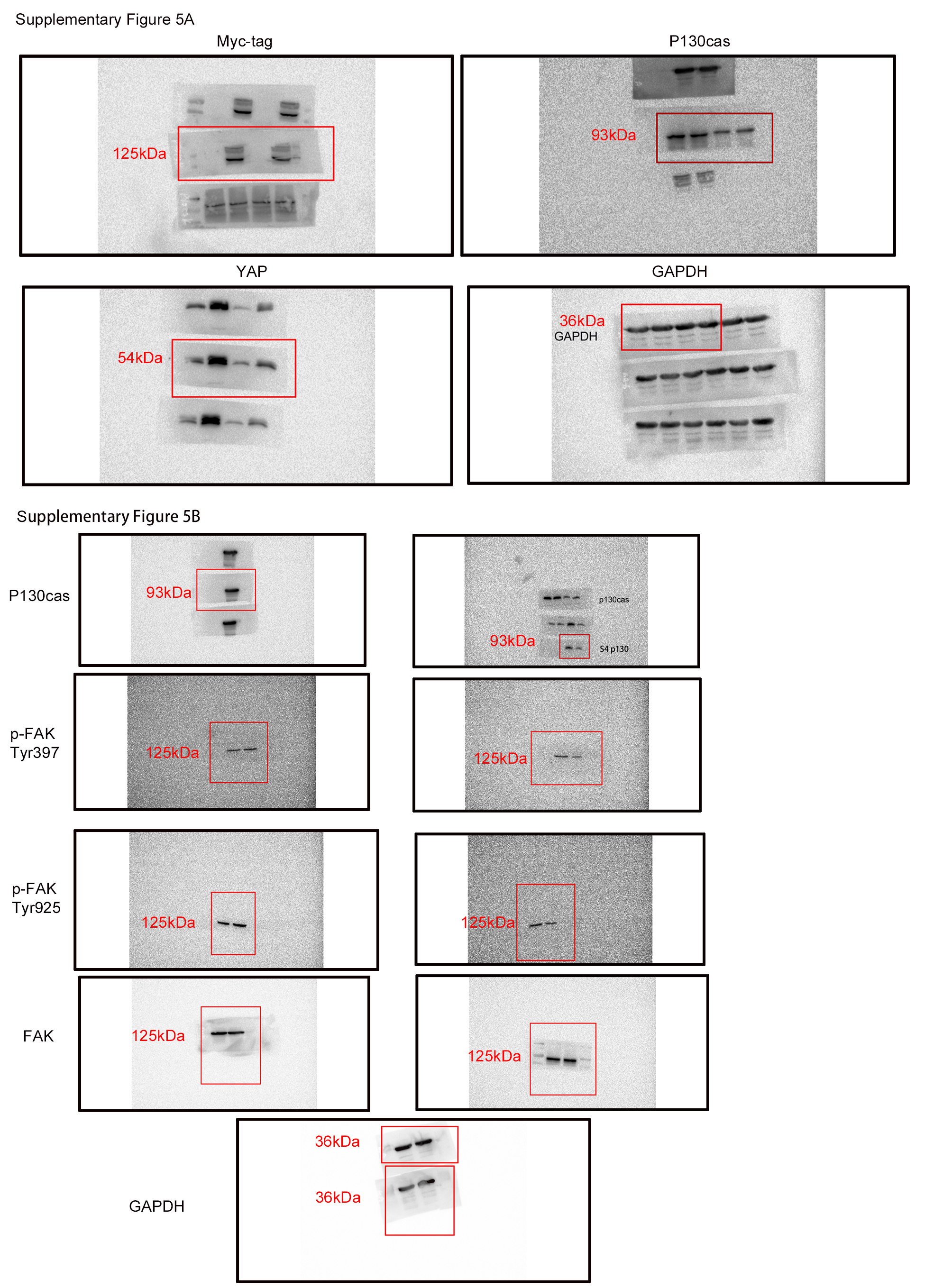


Supplementary Figure 5D-H-I-J


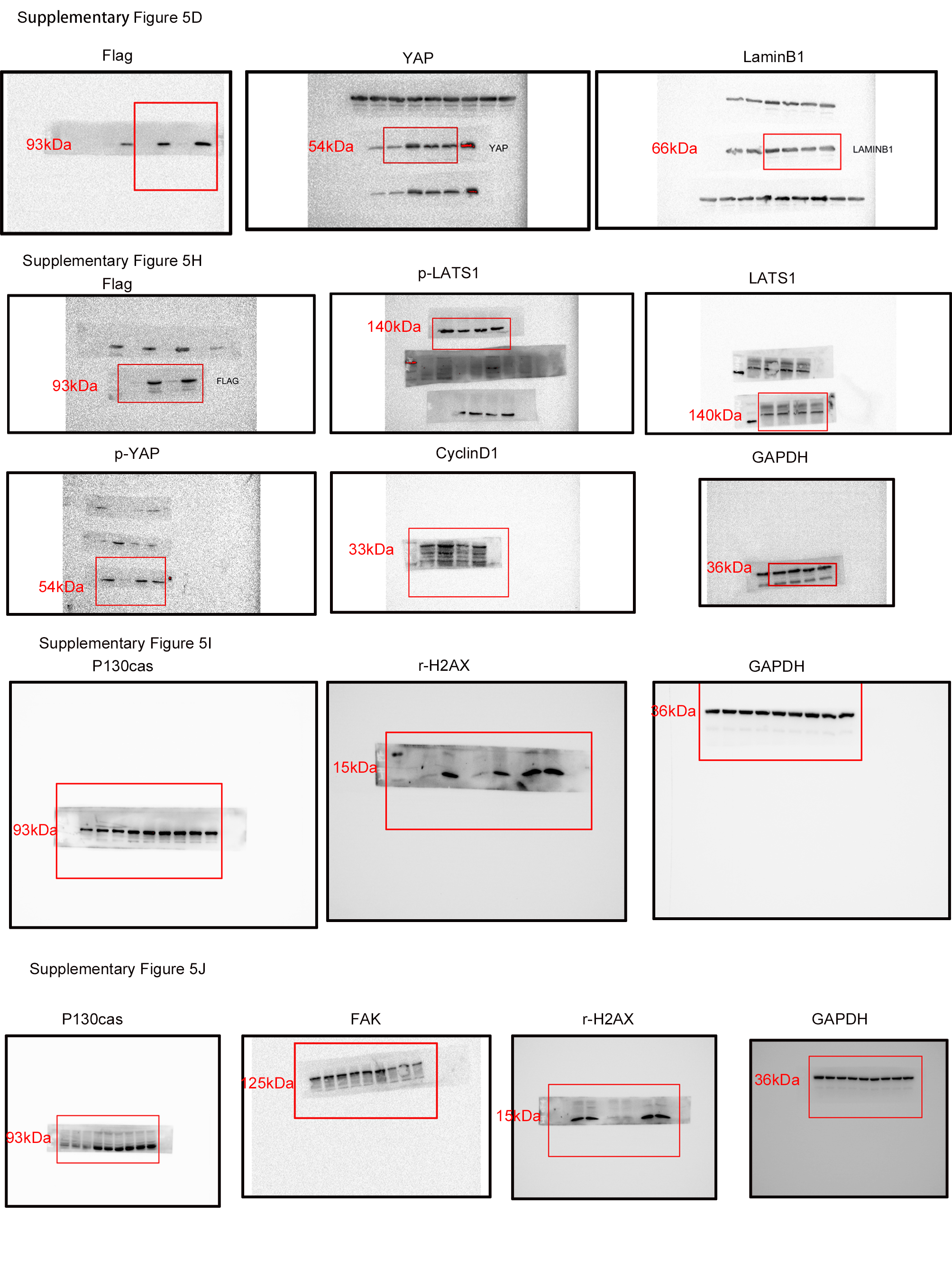


Supplementary Figure 6


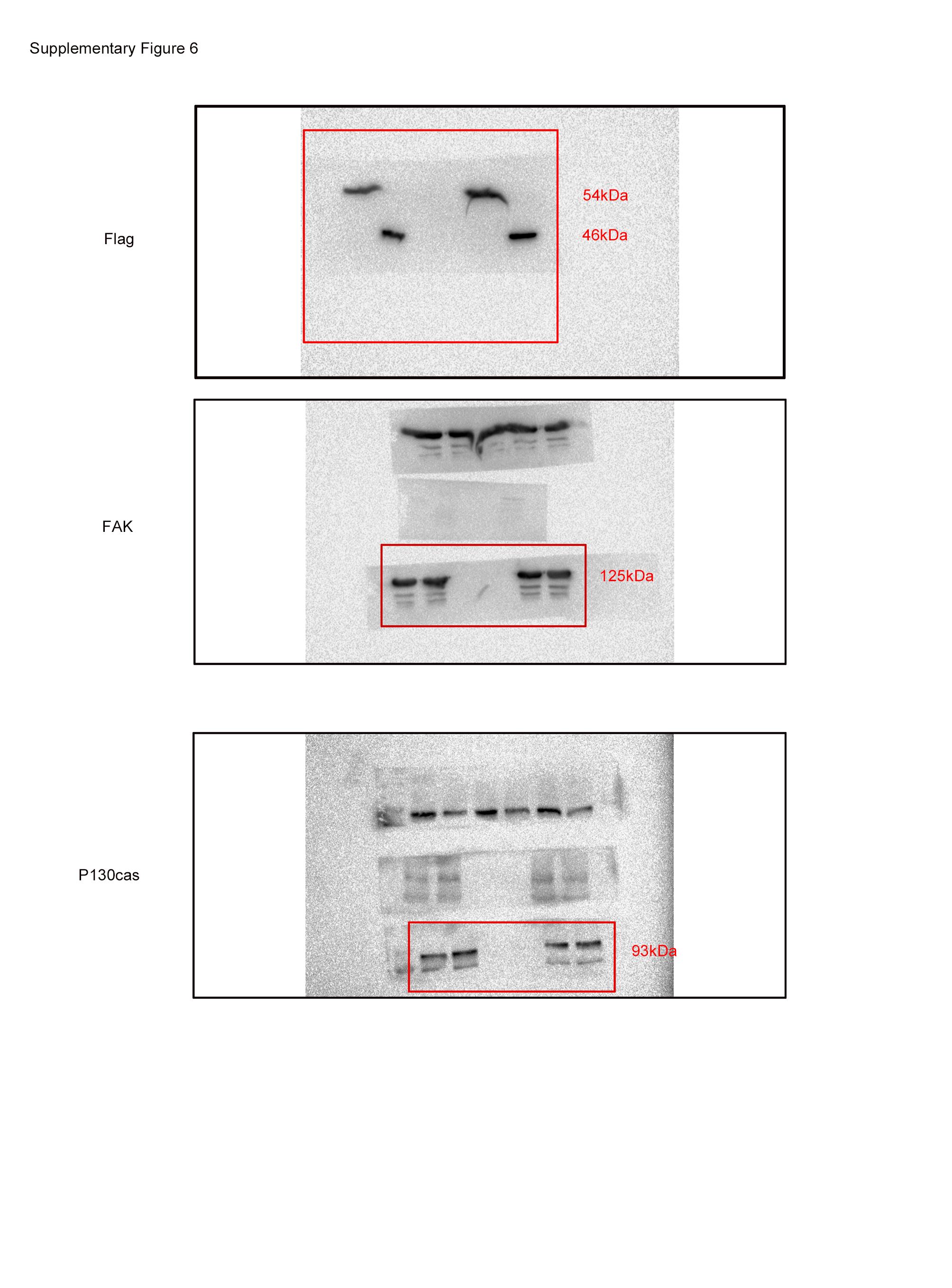

Supplement: Supplementary file 3 — Original Data File [file 41419_2022_5224_MOESM3_ESM.docx]
